# Role of Fatty Acids in Milk Fat and the Influence of Selected Factors on Their Variability—A Review

**DOI:** 10.3390/molecules23071636

**Published:** 2018-07-04

**Authors:** Oto Hanuš, Eva Samková, Ludmila Křížová, Lucie Hasoňová, Robert Kala

**Affiliations:** 1 Dairy Research Institute Ltd., 16000 Prague, Czech Republic; 2 Department of Food Biotechnologies and Agricultural Products´ Quality, Faculty of Agriculture, University of South Bohemia, 37005 České Budějovice, Czech Republic; samkova@zf.jcu.cz (E.S.); faktotum@centrum.cz (L.H.); KalaRobert@seznam.cz (R.K.); 3 Department of Animal Nutrition, Faculty of Veterinary Hygiene and Ecology, University of Veterinary and Pharmaceutical Sciences Brno, 61242 Brno, Czech Republic; krizoval@vfu.cz

**Keywords:** dairy cow, milk fatty acid profile, breed, season, lactation, nutrition, energy status, feeding, organic system, genetic polymorphism

## Abstract

Fatty acids (FAs) of milk fat are considered to be important nutritional components of the diets of a significant portion of the human population and substantially affect human health. With regard to dairy farming, the FA profile is also seen as an important factor in the technological quality of raw milk. In this sense, making targeted modifications to the FA profile has the potential to significantly contribute to the production of dairy products with higher added value. Thus, FAs also have economic importance. Current developments in analytical methods and their increasing efficiency enable the study of FA profiles not only for scientific purposes but also in terms of practical technological applications. It is important to study the sources of variability of FAs in milk, which include population genetics, type of farming, and targeted animal nutrition. It is equally important to study the health and technological impacts of FAs. This review summarizes current knowledge in the field regarding sources of FA variability, including the impact of factors such as: animal nutrition, seasonal feed changes, type of animal farming (conventional and organic), genetic parameters (influence of breed), animal individuality, lactation, and milk yield. Potential practical applications (to improve food technology and consumer health) of FA profile information are also reviewed.

## 1. Introduction

Fatty acids (FAs) in milk fat are considered to be important nutritional components of the diets of a substantial part of the human population. According to scientific knowledge, they can also affect human health. In the past, FAs have been regarded as having negative impacts on human health; however, in the last ten years this notion has been greatly reassessed. Currently, the impact of milk fat on human health is thought of in a much more positive way than it was in previous periods [1,2,3,4,5,6,7]. Nevertheless, this area of research remains a very attractive subject for further expansions of our knowledge.

**Figure 1:**
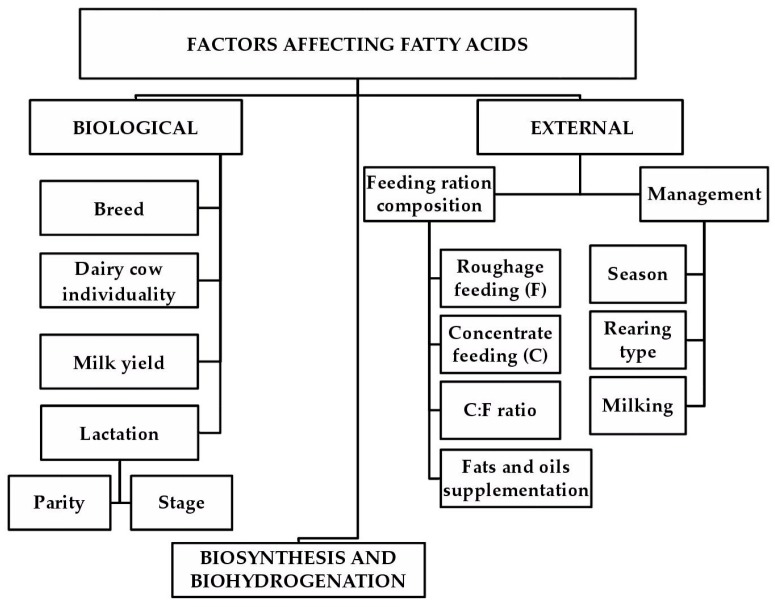
Diagram of the sources of variability in the fatty acid profile of milk.

In the case of dairy farming, the FA profile is also seen as an important factor in the technological quality of raw milk. Therefore, the FA profile has the potential to significantly contribute to the production of dairy products with higher added value. As such, FAs also have an economic importance. Current developments in analytical methods and their increasing efficiency enable the study of milk FA profiles not only for scientific purposes but also in terms of practical technological applications. It is important to study the sources of FA variability in milk, which include population genetics, farming management, and targeted animal nutrition (Figure 1). It is equally important to study the health and technological impacts of FAs.

Efforts to carry out practical improvements of milk FA profiles to benefit consumers are usually driven by two reasons: (1) from a nutritional point of view, a lower proportion of saturated FAs (SFA) and a higher proportion of unsaturated FAs (UFA), especially polyunsaturated FAs (PUFA) n-3, is desirable; and (2) from a usability point of view, higher proportions of UFA are preferred (i.e., easier spreadability of butter is desirable for consumers). However, there are also problems associated with having high UFA content in milk fat, including its lower stability and the accompanying phenomena such as oxidation and possible sensory changes.

Making desirable changes to the FA profile requires a thorough knowledge of the various factors that influence milk fat composition. It is also important to know the extent to which these relevant factors are involved in influencing the FA profile. Some factors affecting the FA profile of milk (such as altitude, breed, lactation order (parity), lactation stage (days in milk), and diet) have been described previously [8,9,10,11,12,13]; nevertheless, these factors continue to be studied because of their wide-range of variation and their large number of possible mutually combined effects. In some studies using multifactorial datasets, the main factors affecting milk FA composition were feeding ration, herd, cow´s individuality, and lactation stage; whereas, breed and parity showed only small effects [10,14,15,16,17]. Although animal factors evidently affect the FA profile of milk fat [18], the main factors are related to dairy cow nutrition [19]. A number of papers showing specific nutritional effects of cow diet on milk FA profiles have been published [20,21,22,23,24,25,26,27,28,29,30,31,32,33]. These results regularly show that increasing the proportion of fresh (pasture) or preserved forage (generally fiber) as compared to grain concentrates and increasing the proportion of oilseeds in feed concentrates as compared to non-oleaginous seeds in dairy cow feeding rations improves the milk FA profile by increasing UFA and rumenic acid (RA; C18:2 *c*9, *t*11; isomer of conjugated linoleic acid (CLA)) content in milk fat.

In-depth knowledge of these factors can also be used to predict milk FA profile. This can be carried out effectively for bulk milk samples based on information about farm practices (especially the composition of dairy cow nutrition and altitude). Good prediction models for SFA and PUFA (determination R^2^ > 0.5), as well as very good models for *trans* isomers of UFA (TFA) (R^2^ > 0.6) have been utilized for this purpose [12].

The aim of this work is to summarize and evaluate the latest findings on factors that contribute to variability in the FA profile of bovine milk fat.

## 2. Development of Indirect Methods for Determining Milk FA Profile

Due to the great nutritional and technological importance of the FA profile for human health and dairy processing practices, the availability of an effective method for determining and controlling milk FA content is important. Sophisticated analytical methods that are considered standard for FA profile verifications are usually very expensive, professionally demanding, and impractical. For example, the primary method is separation by gas chromatography (GC). Therefore, a more effective analytical process is necessary. Over the past twenty years, there have been significant advances in practical hardware and software development for effective routine indirect analyses of milk FAs [34,35,36,37].

Near infrared spectroscopy has been used and evaluated for successful estimation of milk FA profiles [36]. Results of this method proved reliable for the determination of some major FAs and their groups. Coefficients of determination in external validation were good (≥0.88) for SFA, MUFA, UFA, TFA, C6:0, C8:0, C10:0, C12:0, C14:0, C16:0 and C18:1 *c*9 in oven-dried milk, approximate for PUFA, C18:0, vaccenic (VA; C18:1 *t*11) and RA, and poor for linoleic acid (LA; C18:2 n-6), alfa-linolenic (ALA; C18:3 n-3) acid, PUFA n-6, and PUFA n-3. Quantification was more accurate for oven-dried milk, but good results were also obtained for SFA, MUFA, UFA, and C18:1 *c*9 in liquid milk (0.91, 0.89, 0.9, and 0.86, respectively). Lower result reliability (validation determination) was obtained for TFA and RA (0.75 and 0.6, respectively).

Nevertheless, it is technically more effective to use mid infrared spectroscopy with Fourier transformation (MIR-FT) in a flow design apparatus for liquid milk [34,35,37,38]. There are suitable prediction models for FA profile estimation [34,35] and the calibration equations are useful for predicting C12:0, C14:0, C16:0, C16:1 *c*9, C18:1 *c*9, SFA, and MUFA in milk (determination of validation; g·100 mL^−1^ of milk: 0.74, 0.82, 0.82, 0.65, 0.88, 0.94, and 0.85, respectively; g·100 g^−1^ of fat: 0.64, 0.67, 0.5, 0.37, 0.53, 0.63, and 0.52, respectively). In all cases [37], the predictions were of better quality for FAs present at medium to high concentrations (i.e., for SFA and some MUFA with a coefficient of determination in external validation > 0.9). Conversion of FA content expressed in grams per 100 mL of milk to grams per 100 g of FAs was possible with only a small loss of accuracy for some FAs. Calibration correlations between GC and MIR-FT results [38] were higher for SFA and UFA (0.71 and 0.94, respectively; *p* < 0.001); whereas, they were lower for TFA and PUFA (0.59 each; *p* < 0.001). This means that 50.3% and 88.2% of the variability in routine values of SFA and UFA were explainable by variations in reference values. These indirect routine analytical methods are now opening the door to more intensive and effective research studies and quantifications of the sources of variability in milk FA profiles. 

## 3. Effects of Nutrition and Metabolic Aspects of Cattle on Milk FA Profile

### 3.1. Effect of Diet

The amount of milk fat and its composition depends mainly on two processes: lipid metabolism in the rumen and lipid metabolism in the mammary gland. Furthermore, FAs released from body reserves during negative energy balance at early lactation also contribute to the final composition of milk fat [39]. Metabolic processes in the rumen and the composition of rumen microbiota are affected by nutritional factors, especially by the type of forage, forage:concentrate ratio and the associated starch level, use of lipid supplements, and also by the interaction of these factors resulting in changes in duodenal flow and the proportion of each FA [39].

#### 3.1.1. Type of Forage

In many countries, intensive production of milk relies on two feeding strategies: year-round indoor feeding based on preserved feed or seasonal feeding based on grazing during summer combined with indoor feeding during winter. Preserved feed is represented mainly by corn, grass, legume, grass-legume silages or a combination of the above, all supplemented with concentrates. The spectrum of plants used for feeding purposes depends largely on local soil and climatic conditions. Furthermore, the proportion of forage in the diet of dairy cows can range from 50 to 90 percent of dry matter. Diet composition is the main factor that can cause shifts in the microbial diversity of the rumen [40] with subsequent changes in milk FAs. This is because individual categories of bacteria have different lipid metabolism and thus produce higher proportions of specific FAs, such as odd-chain FAs (liquid phase bacteria), C18:1 *trans* isomers (bacteria firmly attached to feed particles), or branched-chain FAs (bacteria loosely attached to feed particles) [41]. Recent research has demonstrated that a shift in rumen microbiota followed by changes to the milk FA profile can occur in response to high-starch diets [42], oil supplementation [43], changes in forage:concentrate ratio [44], or a switch from total mixed ration (TMR) to pasture [45], as also documented in Table 1 comparing pasture-based and silage-based feeding systems. It is clear that regardless of the botanical composition of the pasture, milk fat of grazed cows had the lowest proportion of C16:0 and hypercholesterolaemic FAs (HFA; C12:0 + C14:0 + C16:0) as well as the highest proportions of C18:1 *c*9, VA, RA, and MUFA compared to silage-based feeding [46].

Of the types of silage mentioned in Table 1, a significantly lower proportion of C16:0 (27.6%) as well as a significantly higher proportion of ALA (0.95%), PUFA n-3 (1.20%) and essential FAs (LA + ALA, 2.64%) have been observed in legume silage [46] (as also previously noted, e.g., [47,48]). Furthermore, proportions of the above mentioned FAs are present in even higher proportions in milk from silage-fed cows than in milk from grazed cows, in which the proportion of LA is predominantly low [49,50]. This is similar for cows fed grass silage [20].

According to the literature [24,51], the least favorable FA profile of milk fat was observed in cows fed corn silage-based diets because of the high proportion of HFA (51.8%) and low proportion of C18 FAs (26%), regardless of having the highest recorded proportion of LA (1.92% [46], likely originating from the corn grain [4]).

From the perspective of enhancing the HFA/C18 ratio (see Table 1), grazing represents a good strategy for improving milk fat composition because it increases the proportion of desirable FAs, mainly C18:1 *c*9, RA and *cis*-MUFA, and decreases the proportion of SFA compared to silage-based feeding. Of the preserved feedstuffs, the most suitable seem to be either legume silage or mixed silage [46].

#### 3.1.2. Oilseeds

Using oilseeds in dairy cow diets is a common nutritional strategy used to improve the FA profile of milk fat. Their effect on milk FA composition has been recently reviewed in many studies [39,52,53]. Soybean and rapeseed products are widely and commonly used in many countries as excellent sources of high-quality protein and energy. However, these two feeding components differ in FA profile. While soybean products represent sources rich in LA (52.5%) and lower in C18:1 *c*9 (20.3%) and ALA (6.8%), rapeseed products, in general, are a good source of C18:1 *c*9 (more than 50%) with lower amounts of LA (20.6%) and ALA (8.9%) [54]. As documented in Table 1, soybean products improve proportions of LA and also slightly ALA, thus increasing the essential FAs PUFA n-6 and PUFA n-3 compared to rapeseed products or mixes of oilseeds. On the other hand, as documented in many studies, rapeseed improved the FA profile of milk by increasing the proportion of C18:1 *c*9 and RA and by decreasing C14:0 and C16:0 (this was also true when feeding mixes of oilseeds) [55]. Based on the HFA/C18 ratio (see Table 1), supplementing diets with rapeseed products seems to be better than supplementation with soybean products or mixes of oilseeds because rapeseed products have a greater impact on the proportion of desirable FAs, mainly C18:1 *c*9, VA, and RA.

### 3.2. Effects of Dairy Cow Farming System (Organic Versus Conventional)

As the most important factor affecting the variability of milk FA profile is dairy cow nutrition, comparison of organic (OS) and conventional (CS) farming systems seems to be very important. OS is characterized by grazing, with a reduced amount of conserved forage (but a higher ratio of this type of feed than concentrates) and a small proportion of grain concentrates compared to CS. The main reason for the differences between organic and conventional milk is predominantly in terms of nutritionally desirable components, rather than a genotypic effect [59,60].

In terms of nutrition, comparing milk fat of organic and conventional milk is interesting. Statistically significant differences between OS and CS were only found in the proportions of PUFA and TFA. Both groups of FAs were found to have higher proportions in organic milk [46,61] than in conventional milk. These differences were mainly observed for PUFA n-3 [62,63,64]. The increase of PUFA n-3 in organic milk is due to a significantly higher ALA proportion (0.51, 1.85%; Table 2). In contrast, due to the reduced LA proportion (2.74, 2.39%), milk fat of organic milk had a total reduction in PUFA n-6 (2.54, 2.03%).

In extensive herds (low-input non-organic farms) with mostly pasture-based feeding (94% of dry matter in feed ration) and no conserved forage, the increase in RA, ALA and MUFA was even more pronounced than in OS and CS where pasture was combined with conserved feeding [66]. On the other hand, the amount of fresh forage (pasture) did not significantly affect the composition of milk fat [68]. This finding may be due to the type of forage used in the study, as the predominant proportion was ryegrass. Ryegrass, in contrast to botanically diverse grass and pasture growth [49,69], has a small proportion of ALA. Furthermore, increased amounts of natural and fat-soluble antioxidants (alpha-tocopherol and beta-carotene) have been found in the milk fat of extensively reared dairy cows. These antioxidants probably influence oxidative stability, which is beneficial considering the higher proportion of UFA in this milk fat [66].

Similarly, C18:1 *c*9, LA, and ALA, were more abundant in organic milk compared to conventional milk in the relevant study [7]. In particular, LA and ALA were 24% and 50% higher in organic milk, respectively (*p* < 0.05). In contrast, C16:0 and C18:0 were 10% higher in conventional milk (*p* < 0.05). SFAs were 4% higher in conventional milk; whereas, UFAs were 9% higher in organic milk (*p* < 0.05). PUFAs, including the essential FAs n-6 and n-3, were 25% higher in organic milk. Furthermore, the nutritionally desirable FA parameters indicated by the n-6/n-3 and PUFA/MUFA ratios were 33% and 25% higher in organic milk than in conventional milk, respectively. In general accordance with the above-mentioned results, concentrates, corn (or other) silage, hay, and straw decreased the nutritionally desirable FAs such as MUFA, PUFA, PUFA n-3, ALA, and n-6/n-3 ratio; whereas, these feeds increased SFA, PUFA n-6, and LA [60].

Results for MUFA and PUFA show greater consistency, and higher proportions of VA, RA, ALA, and eicosapentaenoic acid (EPA; C20:5 n-3) in organic milk have been reported independent of the country of origin [70].

It is interesting that organic milk usually yields more nutritionally favorable FA profiles (like PUFA, PUFA n-3, ALA and RA) than conventional milk [60,61,71]. This could be hypothetically explained by more arguments which are linked with the increased proportion of fresh forage in dairy cow nutrition [7]: —dairy cow feeding under low-input (pasture) and organic conditions has usually higher UFA proportion [12,25,26,32,39,47,72,73]; —there can be reduced rumen biohydrogenation [7,72,73,74]; —Δ9-desaturase activity can be influenced either positively [7] or mostly negatively in dependence on long-chain UFA intake [73], extent of PUFA n-3 inhibiting activities [75], content of *de novo* and preformed FAs [74] or precursor concentrations [28]. Also, higher polyphenol and terpenoid intake by cows in OS inhibit hydrogenation by microorganisms in the rumen and this can lead to the higher proportions of desirable FAs in organic milk [62,76,77].

In general, up to 44% of milk fat can originate from a cow’s diet. Therefore, management of animal nutrition during lactation is considered to be critical for producing a high quality FA profile in milk fat, more so than any other factors such as cow breed and genotype, age, health, and aspects of lactation [7,62,65,76].

### 3.3. Effect of Lactation and Energy Status

Lactation stage, along with energy balance of dairy cows, has an impact on the FA profile of cow’s milk. Changes in milk FA composition during lactation, particularly at the beginning of lactation, originate from altered activity in pathways of FA derivation (i.e., the diet; *de novo* synthesis in mammary glands; ruminal biohydrogenation; body fat mobilization) [78,79,80,81]. Lactation itself is characterized by alternating cycles of lipolysis and lipogenesis in body stores that allow the cow to meet her energy requirements for milk secretion. The increased energy demands of foetal development and milk secretion are mainly evident in the transition period of lactation [82]. Therefore, cows, like other lactating animals, often enter a negative energy balance (NEB) at the start of lactation [83], approximately during the initial 30 days [84], or even up to 70 to 84 days postpartum (pp) [85]. Mammary function has metabolic priority and thus, in the NEB state, the limited available nutrients in an organism are directed to milk synthesis for survival of offspring [86]. Body reserves (fat, and to a lesser degree, protein) are mobilized [87] through homeostatic regulation [85,88]. This mobilization results in a loss of body condition score (BCS) and live weight [89,90,91] as a physiological mechanism to overcome the energy deficit. Consequently, non-esterified FAs (NEFAs) are released from body fat reserves, with increasing NEFA levels in blood suggesting a shortfall in energy balance [92]. NEFA metabolites are directed into the mammary gland to supply milk triglycerides or utilized in the liver [86,93,94,95]. 

**Figure 2:**
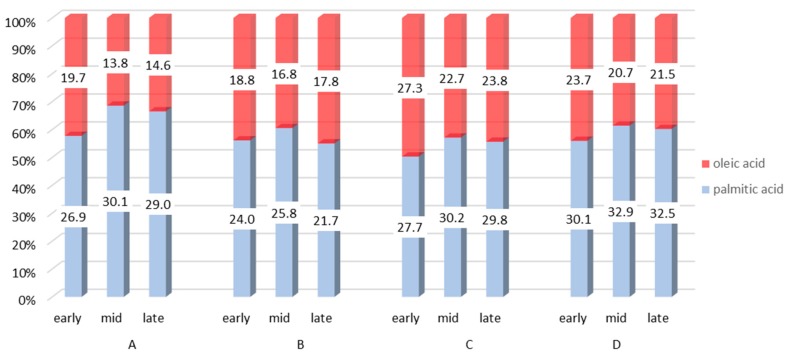
Proportions of palmitic and oleic acids (g∙100 g^−1^ of fatty acids) depending on lactation stage. References: A = [100], B = [101], C = [102], D = [103].

Thus, the beginning of lactation is the most demanding period in terms of energy status and also herd health management [83,86,96,97]. High utilization of energy reserves during this period is reflected in milk fat content [98], namely in the FA composition and mutual ratios between individual FA groups [99]. The general pattern can be described as follows: a high uptake of long-chain FAs by the mammary gland affects *de novo* synthesis of FAs through the inhibition of acetyl coenzyme A carboxylase [8]. Therefore, SFAs, especially C16:0, are at their lowest proportion in week 1 pp, with increasing amounts until 12 weeks pp (as the energy balance improves). On the other hand, MUFAs, mainly represented by C18:1 *c*9, decrease in proportion until week 12 pp [8,78,80,81,100] (Figure 2).

Oleic acid, as the predominant FA in adipocytes, is primarily released through lipolysis during NEB [104,105]. Moreover, adipocytes of high genetic merit cows were found to be more sensitive to lipolysis [106]. An elevated proportion of C18:1 *c*9 in milk fat is a suitable marker for NEB not only in the early lactation period but also in any stage of lactation when fasting and ketosis occur [107]. This was confirmed by a study in which NEB was deliberately induced by feed restriction and it was found that changes in the FA profile follow similar patterns as in the case of NEB in early lactation. Specifically, there is a decreasing proportion of short-chain FAs and an increasing amount of long-chain FAs during NEB. The proportion of PUFA was relatively constant in both postpartum- and feed restriction-induced NEB [81]. 

With improvements in energy balance through progression of lactation or increased feed intake, the FA profile of milk markedly changed [81]. A significantly higher proportion of SFAs, described around day 150 pp, was associated with the later stages of lactation [97], when animals were no longer in NEB. Similarly, the lower proportion of MUFAs around day 150 pp indicates a well-balanced energy intake in cows [99]. Some studies have suggested using measurements of milk FAs, particularly those of long-chain FAs, as indicators of energy status in dairy cows [81,97,107].

According to the aforementioned facts, it is obvious that milk FA composition primarily reflects differential utilization of body fat stores. In this context, the BCS of a dairy cow is a useful tool for indicating energy status. BCS may play a role in regulating appetite and feed intake, thereby affecting milk production and its composition. The optimal calving BCS is 3.0 to 3.25; whereas, a lower calving BCS is associated with reduced milk yield. A BCS ≥ 3.5 is associated with reduced dry matter intake, resulting in decreased milk yield and an increased risk of metabolic disorders [85]. The BCS at calving is positively correlated with milk fat components derived from adipose tissue (long-chain and UFA) and negatively related to short-chain FAs synthesized from ruminal acetate [108,109].

Metabolic status of dairy cows is further affected by parity [110], although studies disagree as to the relationship. Some studies have reported that primiparous cows have greater corporal reserves (BCS) during their postpartum periods than multiparous cows [85,110]. According to other studies, primiparous cows have a lower BCS to begin with and a greater decrease in BCS than multiparous cows [111,112]. Additionally, one study found no difference in BCS based on parity [113]. Due to the high energy requirements for continued body growth [17,113], primiparous cows invest less body reserves into their milk yield compared to multiparous cows. Peak NEFA concentrations occurred at 4 weeks pp for primiparous cows; however, multiparous cows had two peaks—the first at week 1 and the second at week 4 pp. Multiparous cows showed a larger NEB with longer duration than primiparous cows [110].

Considering the health aspects, a more desirable FA composition is observed in cow’s milk at the beginning of lactation (i.e., between days 10 and 30 pp). Three FAs that are referred to as HFA [114] are lowest at the start of lactation but increase as lactation progresses. By contrast, the proportions of C18:1 *c*9 and LA, which are considered to have cardioprotective effects, are present in higher amounts early in lactation [16,81]. RA is another FA with health benefits [115]. According to some studies, stage of lactation has little effect on RA content [10]; however, others have reported an increasing proportion of RA in late lactation [18,116].

In terms of parity, primiparous cows have a nutritionally more desirable FA composition, with a lower proportion of SFA and a higher proportion of UFA and RA [117,118].

## 4. Effects of Genetics and Breeding of Cattle on Milk FA Profile

Use of genetic knowledge to alter milk fat is possible only with these prerequisites: (1) genotypic and phenotypic variation; and (2) accurate estimation of genetic parameters (i.e., heritability, genetic correlation). Fat content is much more variable in comparison to other milk constituents (e.g., protein, lactose). High variability was found not only in fat content, but also in the proportion of FAs and their groups [46,119,120]. Regarding phenotypic differences in individual FAs, variability is higher for UFA than for SFA (Table 3). This is primarily related to the pathway of FA biosynthesis [121,122,123]. Most SFAs are synthesized de novo (in mammary epithelial cells); whereas, most UFAs are obtained from the diet and body fat stores [98].

Finally, knowledge of genetic parameters is important for the design of animal breeding programs and for prediction of selection responses [125]. Evaluation is estimated based on pedigree data; however, the implementation of genetic evaluations for milk FA profile has been limited to a great extent due to the reference method of FA analysis—costly and time-consuming GC [119,120,126]. Use of newer routine methods (MIR-FT) for FA analysis eliminated this drawback and has revealed great potential for genetic analysis of FAs at the population level [120].

### 4.1. Heritability Estimates

The first studies on heritability of FA profiles began in the 1970s [127,128]. These were performed in dairy cows using GC results of FA profiles of very small progeny populations and twins. The results were therefore somewhat limited; however, the studies did increase interest in the topic and over the last decade, especially, many follow-up studies have been published (Table 4). Increasing interest in this topic was initiated by new findings in genetic research and developments in the methods for FA determination (i.e., increased speed and ease of use as well as reduced cost of FA analysis) [119,123,124].

Generally, heritability depends on FA saturation and on carbon numbers of the FAs (Table 4). As such, heritability estimates are mostly higher for SFAs than for UFAs, and an increase in carbon number reflects a decrease in the heritability estimate (higher heritability for short- and medium-chain FAs than for long-chain FA groups) [123,124,129,130]. Consistently, these estimates correspond with the FA biosynthesis mechanism. *De novo* synthesized FAs are influenced mainly by animal factors (breed, parity, stage of lactation); whereas, pre-formed FAs are influenced by feed factors [98]. However, some authors [124,125] have reported slightly higher heritability estimates in PUFA than in MUFA. This difference could be due to the pathway of RA synthesis. RA is produced not only through biohydrogenation of LA in the rumen, but also to a much larger extent (about 80%) by endogenous synthesis from VA by the ∆9-desaturase enzyme in the mammary gland and other tissues [131]. Therefore, there is a noticeable effect of genetic variance.

As shown in Table 4, there are considerable differences in heritability estimates between some studies. The authors mostly agree that this variation in heritability estimates can be explained by the following: (1) the units used to express FA concentrations (i.e., g·100 g^−1^ of milk vs. g·100 g^−1^ of FA vs. g·100 g^−1^ of fat); (2) the methods used for FA analysis (reference GC vs. routine MIR-FT); (3) the models used for estimation of heritability (genome- or pedigree-based analyses); and (4) the database structure. These database structures are determined by the relative genetic and environmental variances because each have a different design given by the number of herds (breeds, sires, cows) or by the number of samples available for analysis [101,122,132,133]. Heritability estimates also differ by parity or stage of lactation [125] and the feeding system [122].

Some studies [121,122,126] provide evidence that additive genetic effects are responsible for a significant proportion of the phenotypic variation in ∆9-desaturase activity in dairy cows. Heritability estimates were comparable to the heritability of milk yield. Desaturase activity could, therefore, be used in future breeding programs to improve the FA profile of milk fat by increasing the proportions of MUFA and RA and by decreasing the SFA proportion.

### 4.2. Genetic and Phenotypic Correlations

Heritability estimates for FAs are usually higher for FAs expressed in 100 g of milk or in g per day, rather than in 100 g of fat or 100 g of FA [132,134]. This demonstrates the considerable effect of milk yield and fat content [135]. Both parameters (milk yield, fat content) are strongly influenced by breed, cow´s individuality, parity, and stage of lactation. Thus, it seems the relationships between milk performance parameters and individual FAs could be useful for understanding the effects of biological factors and, to a certain extent, for determining the extent of the differences [46].

Overall, results for genetic correlations indicate that cows are genetically predisposed to produce more SFA when fat content increases [120]. On the other hand, fat content can be negatively correlated with UFA, suggesting selection for increased fat content will decrease these FAs [123,135] (Table 5). 

FAs were negatively correlated with milk yield and protein yield, but positively correlated with fat yield, fat content, and protein content [119]. Very similar genetic correlations have been reported [120] for FAs depending on FA saturation.

It has been noted [124] that correlations of FAs with milk yield vary across days in milk. Many FAs have genetic correlations with milk yield that are close to zero at the beginning of lactation; yet, as lactation progresses, these correlations become more negative. These differences mean that selecting for milk yield at different stages of lactation could have variable effects on FA content. This may be affected by the energy balance status of cows in early lactation because the highest correlations with lactation were observed for C18:1 *c*9, which is an indicator of body fat mobilization (0.42 at 5 days in milk and −0.40 at 230 days in milk).

Similar to heritability estimates, genetic correlations vary depending on parity [119], with the highest correlations found for first lactation and the lowest for third lactation (for all FAs and their groups).

It should be noted that it is also possible to apply genetic information in attempts to make changes to fat composition, even though UFA expressed a strong negative phenotypic correlation and a weak genetic correlation [126].

### 4.3. Gene Polymorphism

A large number of genes participate in milk fat biosynthesis (Table 6). Despite the availability of whole-genome association studies [138,139,140,141,142,143], our knowledge of the role of various genes is not yet complete. Therefore, identified genes are called candidate genes. Candidate genes for milk fat biosynthesis are associated with different activities, such as: acetate and FA activation and intra-cellular transport (*ACSS2*, *FABP3*), synthesis and desaturation of FAs (*FASN*, *SCD1*), triacylglycerol synthesis (*AGPAT6*, *DGAT1*), regulation of transcription (*SREBF1*, *PPARGC1A*), and others [144]. Polymorphisms in these genes may affect fat content or FA profile and consequently the technological properties of milk fat. 

In relation to FA profile, polymorphisms of the *DGAT1* (K232A) and *SCD1* (A293V) genes have been thoroughly studied [53,145,146]. Genetic variance explained by *DGAT1* is lower (3–15%) than the variance explained by the *SCD1* (6–52%) polymorphism [147].

### 4.4. Effects of Cattle Breed on Milk FA Profile

Cattle breed may affect the FA profile of milk [11,18,30,35,134,148,149,150,151]; however, its influence has sometimes been shown to be limited [17,30]. Ever since the early days of cattle domestication, but especially over the last 150 years, gene homogeneity within milked breeds has increased. In this way, genotypic and consequently phenotypic differences have become more pronounced between breeds for desirable properties (exteriors, metabolic, etc.), including differences in the FA profile. However, according to a summary of past research results, the influence of breed on FAs can be considered a minor effect compared to the practical influences associated with cow diet. A qualified estimate would be able to evaluate the breed effect on FA variability as 20% compared to about 55% that can be attributed to nutritional and feeding performance, while other effects (25%) are related to lactation factors (for instance).

Some papers have described significant milk FA profile differences between cattle breeds [35,134,149,150,151]. The most often studied milked dairy cattle breeds were Holstein (H), Jersey (J), Simmental, Brown Swiss (BS), Ayrshire (A), and Montbéliarde (M) [10,49,69,149,152,153,154,155,156,157,158]. These results have been summarized [18] as FA profiles for breeds H, J, BS, A and M. For instance, for MUFA, there were mean variation ranges (in g·100 g^−1^ of FAs) as follows (for the same order of breeds as listed in the previous sentence): 25.0–39.9; 20.9–22.5; 20.9–29.7; 21.5–24.8; and 16.1–22.0. The same figures for PUFA were: 3.3–4.9; 3.2–3.5; 2.8–4.7; 2.5–4.3; and 2.6–3.2.

In a recent study from an organic farm, Simmental milk had, in the total content of FAs, a significantly higher content of C12:0, C16:1 *c*9, C17:1 *t*9, LA, ALA, C20:1 *c*9, C20:4 n-6, PUFA, and UFA, as compared to Holstein-Friesian (HF) milk. Additionally, Simmental milk had a lower amount of C15:0, C18:0, C20:0, C22:0, and CLA. Compared to HF milk, the PUFA/SFA and UFA/SFA ratios in the Simmental milk were significantly higher; whereas, the thrombogenic index and the LA/ALA ratio were significantly lower [151].

Some minor local breeds have also been studied with regard to milk FA profile [159]. The Polish Red and White breed had a relatively lower content of nutritionally controversial C14:0 and C16:0 as compared to milk of other breeds. Furthermore, their milk proved to be an excellent source of VA and RA, especially during the grazing season.

In general, it is possible to state that local cattle breeds tend to have a better milk fat composition with respect to desirable FAs than breeds that are more efficient in terms of dairy yield. However, these breeds are more often kept under extensive conditions (more often OS), which is also related to a typical variation in dairy cow diet—the proportion of ingredients in the feeding ration is more in favor of forage (fresh or preserved) compared to grain concentrates than under intensive conditions. There have not yet been enough experiments on breed differences in milk FA profile that have been carried out on cows with the same nutritional conditions (for instance Holstein versus a local extensive breed). Of course then, it is possible that a portion of these breed FA differences is primarily defined by dietary sources.

The variability of FAs in bovine milk due to genetic factors and FA profile differences among cattle breeds have been well-studied [35,134,149,150,172]. Often, significant differences between breeds have been found, as well as acceptable genetic correlations and heritability coefficients for major FAs and their groups. The moderate heritability estimates for major FAs observed in these studies may suggest a genetic effect. Therefore, using genetic information to improve the nutritional quality of milk fat based on FA profiles might be possible. Based on these findings, FA profile data may be useful as a marker for genetic improvements (by breeding, selection) of bovine milk fat nutritional quality (i.e., to increase proportions of FAs that are desirable in terms of human nutrition).

## 5. Indices for Evaluation of Milk Fat Quality

Because it is difficult to evaluate the nutritional and also technological value of milk fat from the content of individual FAs, some ratios or indices have been proposed. First, these indices were used primarily to evaluate the negative effect of C12:0, C14:0, and C16:0 on human health. Formulas for calculating the atherogenic (AI) and thrombogenic (TI) indices as well as a modified calculation of S/P were proposed by Ulbricht and Southgate [173]:

AI = (C12:0 + 4 × C14:0 + C16:0)/(∑MUFA + ∑(n-6) + ∑(n-3));(1)

TI = (C14:0 + C16:0 + C18:0)/((0.5 × ∑MUFA + 0.5 × ∑(n-6) + 3 × ∑(n-3)) + (∑(n-3)/∑(n-6)));(2)

S/P = (C14:0 + C16:0 + C18:0)/(∑MUFA + ∑PUFA).(3)

Furthermore, the hypocholesterolaemic/hypercholesterolaemic ratio (HH) is calculated according to Santos-Silva et al. [174]:

HH = (C18:1 n-9 + C18:2 n-6 + C20:4 n-6 + C18:3 n-3 + C20:5 n-3 + C22:5 n-3 + C22:6 n-3)/(C14:0 + C16:0).(4)

More recently, Chen et al. [175] developed the health-promoting index (HPI), which is the inverse of the AI:

HPI = (∑MUFA + ∑PUFA)/(C12:0 + 4 × C14:0 + C16:0).(5)

The activity of Δ9-desaturase is essential in the process of UFA synthesis. This enzyme catalyzes the introduction of a *cis*-double bond between carbons 9 and 10 of SFAs with a chain length of 10 to 18 carbons. During this process, specific medium- and long-chain SFAs are converted into the corresponding MUFAs [10]. Although the enzyme can catalyze the conversion of C10 to C18 SFAs, its effect on these FAs is not equal, mainly favoring the conversions of C16:0 into C16:1 *c*9 and C18:0 into C18:1 *c*9 [98,176]. That is why those FAs are included in the calculation of desaturation indices (DI) that are defined as ratios of the FAs dependent on the activity of this enzyme (*c*9 unsaturated FAs) and can be calculated on the basis of product/substrate [177]; substrate/product [178], or product/(substrate + product) [10] as in the following formulas:

DI = C14:1 *c*9/(C14:0 + C14:1 *c*9), (6)

DI = C16:1 *c*9/(C16:0 + C16:1 *c*9), (7)

DI = C18:1 *c*9/(C18:0 + C18:1 *c*9), (8)

DI = C18:2 *c*9,*t*11/(C18:1 *t*11 + C18:2 *c*9,*t*11). (9)

Although it is possible to calculate DI in different ways, it should be noted that the C14:1 *c*9/(C14:0 + C14:1 *c*9) ratio has been suggested as the best indicator for Δ9-desaturase activity [177] because C14:0 in milk fat is almost exclusively derived from de novo synthesis in the mammary gland and thus almost all C14:1 *c*9 is likely to be the product of Δ9-desaturase activity [14]. Furthermore, a general desaturation index that includes all variables from the above mentioned formulas has been suggested [179]:

DI = 100 × [(C14:1 *c*9 + C16:1 *c*9 + C18:1 *c*9 + C18:2 *c*9,*t*11)/(C14:1 *c*9 + C16:1 *c*9 + C18:1 *c*9 + C18:2 *c*9,*t*11 + C14:0 + C16:0 + C18:0 + C18:1 *t*11)]. (10)

All the abovementioned indices together with PUFA/SFA and n-6/n-3 PUFA ratios are widely used to evaluate the nutritional value of milk fat. It is supposed that milk fat with high AI and TI values may be more likely to contribute to the development of atherosclerosis or coronary thrombosis in humans; whereas, milk with high HPI and HH ratios may have a protective effect against cardiovascular diseases [31]. Although a higher proportion of PUFA in milk fat is desirable from the perspective of human health, these can influence the technological properties of milk fat either positively (improved spreadability) or negatively (increased susceptibility to oxidation). Thus, further indices have been subsequently proposed such as the peroxidisability index (PI, [180], that represents the degree of unsaturation of dietary lipids [181] and is used as an indicator of PUFA peroxidation [182]) and the spreadability index (SI, [183], for evaluating the ratio of C16:0 and C18:1 *c*9, which has been shown to be the most accurate indicator of butter hardness [184]). The oxidative stability of milk fat can also be characterized as SFA/UFA ratio.

PI = 0.025 × Mono + Di + 2 × Tri + 4 × Tetra + 6 × Penta + 8 × Hexa(11)

where: Mono, Di, Tri, Tetra, Penta and Hexa represent the weight percentages of monoenoic, dienoic, trienoic, tetraenoic, pentaenoic and hexaenoic FAs, respectively.

SI = C 18:1 *c*9/C 16:0 (12)

(in some studies, e.g., [21], the inverse formula is used for calculation of SI)

As presented in Table 1, AI is influenced by the type of feed. Of the different types of forage, the lowest AI value was found for pasture-based feeding systems followed by legume silage feeding. The highest values were, on the other hand, found in corn-silage feeding systems. In the case of oilseeds, improvement in AI was noted after feeding either mixes of oilseeds or rapeseed products.

As expected, DI was influenced by the type of forage because of greater differences in the concentration of C18:0 and C18:1 *c*9 [46]. The effect of oilseeds on DI was marginal [27,32,33].

Of the possible forages, the best SI of milk fat was found after feeding corn and grass silages, while the highest value was calculated for pasture-based feeding systems. For oilseeds, an increased SI value and thus softer butter fat was produced after feeding rapeseed products, which has also been documented by e.g., [175].

## 6. Relationships among FA Profiles and Other Indicators in Cows

Biological variability in milk metabolic indicators (such as Fas, etc.) can also be explained, in part, by the relationships among physiological, technological, and health milk indicators. Relationships between FAs and their groups and selected milk indicators (bulk milk samples) were studied in Czech Fleckvieh and Holstein cows [185]. The only significant relationship of SFAs was to lactose content (r = 0.29; *p* < 0.05). All relationships of MUFAs to milk indicators were insignificant (*p* > 0.05). Relationships between PUFAs and milk indicators were narrower: fat (0.32; *p* < 0.05); lactose (0.46; *p* < 0.01); milk alcohol stability (0.45; *p* < 0.01); titration acidity (0.34; *p* < 0.01); cheese curd quality (0.43; *p* < 0.01); milk fermentationability (0.53; *p* < 0.001); streptococci count in yoghurt (0.32; *p* < 0.05); and total count of noble bacteria in yoghurt (0.31; *p* < 0.05). Relationships of CLA to selected milk indicators were as follows: fat (0.38; *p* < 0.01); lactose (−0.54; *p* < 0.001); alcohol stability (0.27; *p* < 0.05); and cheese curd quality (0.41; *p* < 0.01). Thus, higher CLA levels were associated with higher fat and lower lactose content, as well as lower alcohol stability. 

Correlation coefficients (>0.3) for the summarized values of lactation, which were calculated in regular milk recording were observed [13] at MUFA with short chain: 0.47 to days in milk, 0.31 to milk yield (kg), 0.35 to fat, and 0.34 to protein total production (both in kg). The most important FAs (in individual milk samples), such as C12:0, C14:0, C16:0, C18:0, and C18:1 *c*9, were not significantly related to either lactation sum or daily production parameters, or to the content of basic components of milk with the exception of C16:0 (30.93 ± 4.81% in milk fat), which has a negative relationship to daily milk (−0.4) and protein production (−0.35). This FA has also been positively associated with fat content (0.44) and negatively associated with lactose content (−0.31). CLA was negatively correlated with daily fat production (−0.41), fat content (−0.27), and fat/protein index (−0.42). In order to better understand and interpret milk FA profiles, knowledge of the relationships between major FAs and their groups and other milk indicators is also important.

In addition to the above mentioned milk indicators, there are also documented associations between the milk FA profile and metabolic disorders such as ketosis or between the milk FA profile and reproduction performance [81,136]. Moreover, interesting associations have been recently described among FAs synthetized in the rumen, methane production, and milk FA content [129,137]. It is evident that the possibility for methane output prediction based on milk FA content should be intensely studied to improve the environmental sustainability and economic profitability of dairy production.

Last but not least, dairy cow nutrition substantially influences the profiles of other body tissues, especially body liquids, in addition to the milk FA profile. Accordingly, an analysis of the percentage (by weight) of FAs in different body tissues [186] was done. Findings revealed that stage of lactation had a significant impact on the content of many FAs in all examined tissues. Parity had no effect on FA composition of blood; whereas, it significantly affected C16:1 *c*9 in the liver as well as C16:1 *c*9 and C18:0 in adipose tissue. Energy-protein supplementation significantly affected the content of most FAs in blood (e.g., C18:1 *t*11 and C18:3 n-3) and liver (C18:3 n-3, CLA, and PUFA n-3 derived from fish oil), but it did not affect the profile of adipose tissue in cows. Therefore, it is necessary to consider these effects when developing methods to control the production of animal raw materials for the food industry.

## 7. Milk FA Profile and Human Health

Milk and dairy products play an important role in human nutrition because they provide not only essential nutrients such as high-quality proteins, fat, lactose and minerals, but also various physiologically active compounds such as vitamins, bioactive peptides, and antioxidants [187,188,189]. 

Milk and some dairy products (butter) have been criticized for the unfavorable FA profile in milk fat. Indeed, bovine milk fat contains on average about 70% SFA, 25% MUFA, and 5% PUFA [42]; whereas, the ideal FA profile, from a human health perspective, should be 8% SFA, 82% MUFA and 10% PUFA [190]. 

The SFAs that play the greatest role in a negative view of milk fat (i.e., C12:0, C14:0, and C16:0), have often been associated with having adverse effects on indicators of cardiovascular risk (e.g., low-density lipoprotein cholesterol level in serum [191]). This is because the consumption of excessive amounts of SFA has been associated with increased risk of cardiovascular disease [192]; however, extensive modern research on the effects of FAs on human health indicates that only a few individual FAs are responsible for the negative health consequences [193]. Thus, the perspective on SFA has only recently changed from a focus on the effects of SFA as a single group to the effects of individual SFAs as well as other FAs present in milk. Continued study and discussion of their specific biological functions and roles in metabolism (see Table 7), along with their interactions is needed.

## 8. Conclusions

At present, there is strong research interest in the nutritional quality and health benefits of food, which is supported by public awareness and an on-going desire to improve our quality of life. Consequently, targeted modification of the FA profile of milk fat is desirable. Developments in analytical methods have played an important role in increasing the efficiency and feasibility of studies on the sources of FA variability. In recent decades, many studies have been devoted to improving milk FA composition by increasing the amount of FA with beneficial effects on human health and appropriate technological properties. Accordingly, knowledge of the important factors affecting milk FA composition and their relationships, including physiological aspects such as rumen fermentation, fat synthesis in mammary gland tissue, and energy status of animals, is essential from both a research and practical point of view. This review has therefore focused on the main sources of FA variability. Breed, animal genetics, metabolic and lactational effects, management, and other factors were mentioned; nevertheless, feeding strategy is undoubtedly considered to be the most efficient way to modify milk FA composition. Some factors are well known—e.g., role of diet and stage of lactation—while others, such as polymorphism, are only beginning to be understood. The possibility of using milk FA profiles as indicators to predict animal health or even methane production levels is currently under active investigation. Further research on sources of FA variability is important for finding effective ways of improving the health benefits and technological quality of milk products through modifications in the FA profile. This will most likely be achieved by targeted changes to feeding and breeding strategies.

## Figures and Tables

**Table 1:** Effects of the types of forage and oilseed on the proportion of fatty acids (FAs; % of total FA) in milk fat *.

	**C14:0**	**C16:0**	**C18:0**	**C18:1 *c*9**	**C18:1 *t*11**	**18:2 n-6**	**C18:2 *c*9,*t*11**	**C18:3 n-3**	**HFA** ^3^	***c*-MUFA** ^4^	***t*-MUFA** ^5^
	**Mean (*n*)**	**Mean (*n*)**	**Mean (*n*)**	**Mean (*n*)**	**Mean (*n*)**	**Mean (*n*)**	**Mean (*n*)**	**Mean (*n*)**	**Mean (*n*)**	**Mean (*n*)**	**Mean (*n*)**
Silage											
- grass	12.4 (11)	32.3 (13)	9.8 (13)	16.1 (13)	1.18 (6)	1.18 (13)	0.54 (6)	0.49 (13)	48.6 (11)	18.5 (10)	3.65 (10)
- legume	11.0 (6)	27.6 (6)	9.9 (6)	17.6 (6)	1.24 (5)	1.69 (6)	0.51 (5)	0.95 (6)	42.1 (6)	19.7 (6)	3.53 (6)
- mix ^1^	11.2 (5)	32.1 (5)	10.3 (5)	18.8 (4)	0.83 (4)	1.43 (5)	0.47 (4)	0.48 (5)	46.6 (5)	18.2 (1)	2.34 (3)
- corn	13.4 (6)	33.2 (8)	7.0 (8)	16.7 (8)	0.92 (7)	1.92 (8)	0.45 (7)	0.39 (8)	51.8 (6)	18.9 (6)	2.30 (1)
Pasture	10.1 (18)	25.0 (20)	9.6 (20)	19.7 (20)	3.15 (20)	1.04 (17)	1.30 (20)	0.75 (17)	38.1 (18)	21.1 (13)	4.25 (7)
Oilseeds											
- soybean	11.2 (4)	29.4 (4)	10.8 (4)	16.8 (4)	0.99 (3)	3.01 (4)	0.66 (4)	0.45 (4)	43.9 (4)		
- rapeseed	10.8 (6)	28.5 (6)	12.5 (6)	21.2 (6)	1.28 (3)	2.26 (5)	0.75 (5)	0.41 (6)	42.2 (6)	25.0 (1)	6.4 (1)
- mix ^2^	11.3 (3)	33.5 (3)	10.4 (3)	21.4 (3)		2.64 (3)	0.55 (2)	0.3 (3)	48.1 (3)		
	**EFA** ^6^	**PUFA n-6** ^7^	**PUFA n-3** ^7^	**C18** ^3^	**SFA/UFA** ^8^	**n-6/n-3** ^7^	**HFA/C18** ^3^	**AI** ^9^	**DI** ^9^	**SI** ^9^	
	**Mean (*n*)**	**Mean (*n*)**	**Mean (*n*)**	**Mean (*n*)**	**Mean (*n*)**	**Mean (*n*)**	**Mean (*n*)**	**Mean (*n*)**	**Mean (*n*)**	**Mean (*n*)**	
Silage											
- grass	1.67 (13)	1.33 (10)	0.51 (10)	27.6 (13)	2.82 (10)	2.77 (10)	1.77 (11)	3.58 (10)	0.62 (16)	0.50 (13)	
- legume	2.64 (6)	2.09 (6)	1.20 (6)	30.1 (6)	2.49 (6)	1.95 (6)	1.41 (6)	2.83 (6)	0.64 (6)	0.64 (6)	
- mix ^1^	1.91 (5)	1.97 (4)	0.68 (4)	30.3 (4)	2.89 (1)	2.98 (4)	1.59 (4)	3.46 (4)	0.65 (4)	0.59 (4)	
- corn	2.31 (8)	2.43 (5)	0.43 (5)	26.0 (8)	3.03 (6)	6.21 (5)	2.09 (6)	3.82 (5)	0.70 (8)	0.50 (8)	
Pasture	1.79 (17)	1.56 (14)	0.99 (14)	30.5 (17)	2.36 (10)	1.69 (14)	1.33 (15)	2.45 (13)	0.67 (20)	0.79 (20)	
Oilseeds											
- soybean	3.45 (4)	4.08 (2)	1.64 (2)	32.5 (4)	2.24 (4)	2.49 (2)	1.45 (4)	2.52 (4)	0.67 (4)	0.75 (4)	
- rapeseed	2.29 (6)	2.17 (5)	1.02 (5)	37.3 (6)	1.79 (6)	1.71 (5)	1.20 (6)	2.17 (6)	0.67 (6)	0.92 (6)	
- mix ^2^	2.95 (3)	2.88 (3)	0.76 (3)	35.1 (3)	2.1 (2)	3.79 (3)	1.39 (3)	1.90 (3)	0.68 (3)	0.67 (3)	

* Values for forages calculated from 15 publications [46], values for oilseeds calculated from eight publications [22,27,32,33,55,56,57,58]; **^1^** mix from various proportions of corn and grass silages; ^2^ mix of oilseeds (rapeseed + soybean or rapeseed + sunflower); **^3^** HFA—hypercholesterolaemic FAs (∑ C12:0, C14:0 and C16:0); ^3^ C18—(∑ C18:0, C18:1 *c*9, 18:2 n-6 and 18:3 n-3); ^4^ *c*-MUFA—*cis*-isomers of monounsaturated FAs (∑ C14:1 *c*9, C16:1 *c*9 and C18:1 *c*9); ^5^ *t*-MUFA—*trans*-isomers of monounsaturated FAs C18:1, including C18:1 *t*11; ^6^ EFA—essential FAs (∑ C18:2 n-6 and C18:3 n-3); ^7^ PUFA—polyunsaturated FAs, n-6 and n-3; ^8^ SFA/UFA—saturated/unsaturated FA ratio; ^9^ AI—atherogenic index [(C12:0 + 4 × C14:0 + C16:0)/(∑MUFA + ∑(n-6) + ∑(n-3))], DI—desaturation index [C18:1 *c*9/(C18:0 + C18:1 *c*9)], SI—spreadability index [C18:1 *c*9/C16:0].

**Table 2:** Proportions of some fatty acids (FAs) and their groups in bovine milk fat (g 100 g^−1^ of FA) depending on rearing type *.

	Conventional Herds		Organic Herds	
	Mean	*n*	Mean	*n*
C18:2 n-6	2.74	2	2.39	3
C18:3 n-3	0.51	6	1.85	8
C18:1 *t*11	1.82	5	2.74	7
C18:2 *c*9,*t*11	0.64	9	0.91	11
SFA ^1^	68.2	7	68.3	9
MUFA ^2^	26.8	7	27.0	9
PUFA ^3^	4.39	7	4.91	9
PUFA n-6 ^3^	2.54	6	2.03	7
PUFA n-3 ^3^	0.76	4	0.87	4
S/U ^4^	2.19	7	2.17	9
n-6/n-3 ^3^	4.29	5	2.65	6

* Means and numbers of cases from six publications: [62,63,64,65,66,67]; ^1^ SFA—saturated FAs; ^2^ MUFA—monounsaturated FAs; ^3^ PUFA—polyunsaturated FAs, n-6 and n-3 series and their n-6/n-3 ratio; ^4^ S/U—ratio between saturated and unsaturated FAs.

**Table 3:** Mean and coefficient of variation (CV; %) for milk yield, content of fat, protein, and lactose (g·100 g^−1^ of milk), and groups of fatty acids (FAs) ^1^.

	Holstein (Belgium) [124]		Holstein (Italy) [120]		Holstein (Brazil) [125]	
	Mean	CV	Mean	CV	Mean	CV
Milk yield (kg/day)	23.1	25.9	31.6	28.5	34.2	29.5
Fat (g·100 g^−1^)	3.96	13.7	3.70	18.9	3.45	21.7
Protein (g·100 g^−1^)	3.34	9.7	3.40	11.8	3.05	9.9
Lactose (g·100 g^−1^)					4.60	5.2
Groups of FA (g·100 g^−1^ of milk (g·100 g^−1^ of fat))						
SFA	2.79 (74.17)	16.5	2.58 (73.40)	20.5	2.23 (68.04)	22.7
UFA	1.31 (34.79)	17.3	1.11 (31.58)	21.6	1.03 (31.43)	28.8
MUFA	1.13 (29.98)	18.2	0.91 (25.89)	22.0	0.87 (26.54)	30.8
PUFA	0.17 (4.43)	19.2	0.09 (2.56)	33.3	0.16 (4.88)	30.8

^1^ SFA—saturated FAs; UFA—unsaturated FAs; MUFA—monounsaturated FAs; PUFA—polyunsaturated FAs.

**Table 4:** Overview of heritability, method ^1^, number of samples (cows and sires), breed ^2^, and country ^3^ for selected milk fatty acids (FAs) and their groups ^4^.

Reference	Method	g∙100 g^−1^	Number of			Breed	Country	Heritability ^5^												
			Samples	Cows	Sires			h^2^	MY (kg)	F (%)	FY (kg)	C14:0	C16:0	C18:0	C18:1	CLA	SFA	UFA	MUFA	PUFA
[132]	GC	of FA	592	233	53	H	US	h^2^_IH_	0.11		0.19	<0.001	0.09	0.24	0.06		0.05		0.08	<0.001
[14]	MIR-FT	of fat	52,950	3217	1666	H	BE	h^2^_G_	0.20	0.33		0.15	0.15	0.16	0.17					
[135]	GC	of fat		1918	101	H	NL	h^2^_G_	0.29	0.47	0.29	0.49	0.31	0.19	0.18	0.21				
	GC	of fat		1918	101	H	NL	h^2^_IH_	0.41	0.51	0.39	0.59	0.43	0.23	0.25	0.42				
[101]	GC	of fat		990		H	IT					0.07	0.03	0.08	0.17	0.12			0.14	
[122]	GC	of FA		2408	597	HF	GB		0.35			0.09	0.06	0.04	0.12	0.02	0.14		0.09	0.03
[124]	MIR-FT	of milk	130,285	26,166		H	BE		0.20	0.39	0.17	0.44	0.41	0.23	0.18		0.43	0.22	0.21	0.30
[136]	MIR-FT	of milk	143,332	29,792		H	BE		0.31	0.68	0.29	0.68	0.67	0.60	0.52		0.68	0.60	0.58	0.69
[126]	GC	of fat		371	200	H	DK	h^2^_G_		0.24		0.25	0.14	0.19	0.11	0.19	0.09	0.33	0.34	0.28
[133]	GC	of FA		371		H	DK	h^2^_G_				0.16	<0.001	0.14	<0.001					
	MIR-FT	of FA		371		H	DK	h^2^_G_				0.17	0.36	0.33	0.07					
[120]	MIR-FT	of milk	72,848	17,873	1235	H	IT	h^2^_G_	0.10	0.20							0.25	0.07	0.08	0.08
	MIR-FT	of milk	72,848	17,873	1235	H	IT	h^2^_IH_	0.14	0.24							0.29	0.09	0.10	0.15
[137]	GC	of fat		339		H	DK	h^2^_P_				0.16	0.21	0.11	0.07	0.13				
	GC	of fat		339		H	DK	h^2^_G_				0.08	0.17	0.17	0.02	0.18				
[125]	MIR-FT	of milk	36,457	4203	226	H	BR	h^2^_P_					0.26	0.13	0.07		0.25	0.08	0.07	0.11
	MIR-FT	of milk	36,457	4203	226	H	BR	h^2^_G_					0.26	0.14	0.07		0.25	0.08	0.07	0.11
[129]	MIR-FT	of milk	241,236	33,555		H	BE	h^2^_G_				0.42	0.38	0.19	0.15		0.40	0.20	0.19	
[123]	MIR-FT	of fat	612,321	132,731		H	DK	h^2^_IH_				0.09	0.14	0.11	0.13		0.15		0.15	0.08
	MIR-FT	of fat	95,920	21,967		J	DK	h^2^_IH_				0.07	0.16	0.09	0.10		0.10		0.10	0.11

^1^ MIR-FT—infrared spectroscopy in mid-range with Fourier transformation; GC—gas chromatography; ^2^ H—Holstein; HF—Hereford; J—Jersey; ^3^ BE—Belgium; BR—Brazil; DK—Denmark; GB—Great Britain; IT—Italy; NL—Netherlands; US—the United States; ^4^ CLA—conjugated linoleic acid; SFA—saturated FAs; UFA—unsaturated FAs; MUFA—monounsaturated FAs; PUFA—polyunsaturated FAs; ^5^ h^2^_G_—genomic; h^2^_IH_—intraherd; h^2^_P_—pedigree; heritability is estimated based on linear models; the estimate of H^2^_G_, H^2^_IH_, H^2^_P_ is different by the number of variables involved (σ^2^_A_, σ^2^_E_, σ^2^_PEAL_, σ^2^_PEWL_, etc.); MY—milk yield; F—fat content; FY—fat yield.

**Table 5:** Genetic and phenotypic correlations (r) ^1^ between fat content or milk yield and selected fatty acids (FAs) and their groups.

Reference	r ^1^	C14:0	C16:0	C18:0	C18:1	CLA	SFA	MUFA	PUFA
	Fat Content (g∙100 g^−1^)								
[121]	G	−0.43	0.65	0.01		−0.58			
	P	−0.27	0.43	0.08		−0.32			
[123]	G	0.06	0.17	−0.14	−0.26		0.34	−0.33	−0.26
	P	−0.04	−0.05	0.02	−0.04		0.09	−0.08	−0.07
[125]	G		0.98	0.86	0.80		0.99	0.79	0.52
	P		0.90	0.82	0.81		0.95	0.83	0.71
[101]	G	−0.40	0.74	0.28	0.02	−0.55		0.01	
[135]	G	−0.43	0.65	0.01	−0.63				
[119]	G	0.84	0.88	0.71	0.64		0.91	0.72	0.69
	**Milk Yield (kg∙day^−1^)**								
[125]	G		−0.35	−0.28	−0.29		−0.36	−0.32	−0.38
	P		−0.09	−0.06	−0.01		−0.06	−0.03	−0.03
[135]	G	0.30	−0.50	0.15	0.32				
[119]	G	−0.34	−0.33	−0.30	−0.31		−0.36	−0.35	−0.37

^1^ Correlation coefficient; G—genetic; P—phenotypic; CLA—conjugated linoleic acid; SFA—saturated FAs; MUFA—monounsaturated FAs; PUFA—polyunsaturated FAs.

**Table 6:** Overview of selected candidate genes coding enzymes that affect bovine milk fat and fatty acids (FAs).

Reference	Gene	Gene Name	BTA ^1^	Polymorphism	Associated with: ^2^
[160]	*FABP3*	*fatty acid-binding protein 3*	2	A/G transition	fat (↑) and protein (↑) content
[161]	*LEP*	*leptin*	4	A80V	milk yield (↑)
[162]	*ABCG2*	*ATP binding cassette, subfamily G, member 2*	6	Y581S	milk yield (↑), fat (↑) and protein (↑) content
[163] [164]	*PPARGC1A*	*peroxysome proliferator-activated receptor gamma, coactivator 1 alpha*	6	T19C A968C	fat yield (↑) milk yield (↑), protein content (↑)
[141]	*ACSS2*	*acyl-CoA synthetase, short-chain family, member 2*	13	n.a.	activation and intracellular channeling of FAs
[165] [121]	*DGAT1*	*diacylglycerol O-acyltransferase 1*	14	K232A	fat content (↑) C16:0 (↑), C14:0 (↓), C18u (↓), CLA (↓)
[139]	*ACLY*	*ATP citrate lyase*	19	n.a.	biosynthesis of milk fat (especially C14:0)
[166]	*FASN*	*fatty acid synthase*	19	T1950A	fat content (↑), C14:0 (↑)
[167]	*GH*	*growth hormone*	19	GH4.1 GH6.2	milk (↑), fat (↑) and protein (↑) yield
[168]	*STAT5A*	*signal transducer and activator of transcription 5A*	19	A9501G	milk yield (↑), protein content (↑)
[169]	*SREBF1*	*sterol regulatory element binding transcription factor 1*	19	L852P	haplotype H1 – effects on C12:0 (↓) and C14:0 (↓)
[170]	*GHR*	*growth hormone receptor*	20	GHR4.2	milk yield (↑)
[147]	*SCD1*	*stearoyl-CoA desaturase 1*	26	A293V	indices C10 (↓), C12 (↓), C14 (↓), C16 (↑), C18 (↑), CLA (↑)
[171]	*AGPAT6*	*1-acylglycerol-3-phosphate O-acyltransferase 6*	27	n.a.	fat content (↑)

n.a.—not available ; ^1^ BTA—*Bos taurus* autosome; ^2^ C18u—unsaturated C18; CLA—conjugated linoleic acid; ↑—an increase; ↓—a decrease.

**Table 7:** Effects of selected fatty acids (FAs) on human health.

FA	Role	References
C4:0	- beneficial effect on the intestinal flora and human gastrointestinal wall primarily by acting as a direct source of energy for colonocytes - one of the factors preventing progression of colorectal cancer and mammary cancer - inhibition of cell growth, promotion of differentiation, and induction of apoptosis in various human cancer cell lines - may prevent the invasion of tumors via inhibitory effects on urokinase - seems to exert broad anti-inflammatory activity by affecting immune cell migration, adhesion, and cytokine expression, as well as affecting cellular processes such as proliferation, activation, and apoptosis	[2,3,194,195,196,197]
C12:0, C14:0, C16:0, C18:0	- C14:0 and C16:0—increase total blood cholesterol level and increase the risk of cardiovascular diseases - C18:0 and C14:0—increase thrombogenicity and cholesterol level - C12:0, C14:0, and C16:0, are related to an increased risk of atherosclerosis, hyperlipidemia, and low-density lipoprotein cholesterol, obesity and coronary heart disease	[23,60,173,188,198]
BCFA	- BCFA—anti-cancer activity - BCFA—reduced risk of necrotizing enterocolitis in newborns - BCFA—improvement of β-cell function - iso C15:0—anti-cancer properties—induced cell death through apoptosis (in vitro) - iso C15:0—inhibition of tumor growth in mice (in vivo) - iso C15:0—induction of inhibitory effects on T-cell lymphomas in vitro and in vivo in mice	[199,200,201,202,203]
OCFA	- decreased risk of coronary heart disease - decreased risk of type 2 diabetes	[204,205]
TFA	- not confirmed positive relationship between coronary heart disease and TFA of ruminant origin - C18:1 *t*10—a potential negative effect, tendency to increase serum triglycerides (animal models) - C18:1 *t*11—improvement of lipid biomarkers	[206,207,208,209,210,211]
*c*9, *t*11 CLA, *t*10, *c*12 CLA	- reduced tumor growth - decreased risk of coronary heart disease	[189,190,212,213,214,215,216]
C16:1	- considered to be a lipokine released from adipose tissue that acts on distant organs - mixed cardiovascular effects, direct or inverse correlations with obesity, hepatosteatosis, and a significant amelioration or prevention of insulin resistance and diabetes	[217]
C18:1 *c*9 C18:3 n-3	- anti-cancer and anti-atherogenic properties - positive effect on cholesterol level - improvement of immune response (anti-inflammatory effect)	[114,188,218,219,220]
C18:2 n-6	- improves sensitivity to insulin and thus reduces the incidence of type 2 diabetes	[198]
CLnA	- inhibitory effect on cancer cell proliferation and growth of human tumor cells (in vitro) - modification of lipid metabolism (with decreases in adipose tissue mass) in rodent models (in vivo)	[221,222]
C18:1 n-11	- beneficial modifying effect on the fluidity and permeability of cell membranes, regulates their metabolism, and may have anti-cancer properties	[223]
AA EPA	- neutralization of C12:0, C14:0 and C16:0 by increasing high-density lipoprotein cholesterol level - anti-cancer, anti-hypertensive, and anti-inflammatory properties	[6,76,224,225]
DHA	- positive effect on brain cells, which is important during remission of Alzheimer’s disease - anti-cancer, anti-hypertensive, and anti-inflammatory properties	[225,226]

BCFA—branched-chain FAs; OCFA—odd-chain FAs; CLA—conjugated linoleic acid; *c*—*cis*; *t*—*trans*; C18:3 n-3—alfa-linolenic acid; C18:1 *c*9—oleic acid; C18:2 n-6—linoleic acid; CLnA—conjugated linolenic acids, mainly *c*9, *t*11, *c*15, and *c*9, *t*13; *c*15; C18:1 *t*11—vaccenic acid; AA—arachidonic acid; EPA—eicosapentaenoic acid; DHA—docosahexaenoic acid.

## Abbreviations

ALA	alpha linoleic acid
BCS	body condition score
CLA	conjugated linoleic acid
CS	conventional system
EPA	eicosapentaenoic acid
FA	fatty acid
GC	gas chromatography
LA	linoleic acid
MIR-FT	infrared spectroscopy in mid-range with Fourier transformation
MUFA	monounsaturated fatty acids
NEB	negative energy balance
NEFA	non-esterified fatty acids
OS	organic system
PUFA	polyunsaturated fatty acids
RA	rumenic acid (C18:2 *c*9, *t*11)
SFA	saturated fatty acids
TFA	*trans* isomers of polyunsaturated fatty acids
UFA	unsaturated fatty acids
VA	vaccenic acid (C18:1 *t*11)

## References

[1] Nicolosi R.J., Rogers E.J., Kritchevsky D., Scimeca J.A., Huth P.J. (1997). Dietary conjugated linoleic acid reduces plasma lipoproteins and early aortic atherosclerosis in hypercholesterolemic hamsters. Artery.

[2] Parodi P.W. (1997). Cows’ milk fat components as potential anticarcinogenic agents. J. Nutr..

[3] Parodi P.W. (1999). Conjugated linoleic acid and other anticarcinogenic agents of bovine milk fat. J. Dairy Sci..

[4] Dhiman T.R., Nam S.H., Ure A.L. (2005). Factors affecting conjugated linoleic acid content in milk and meat. Crit. Rev. Food Sci. Nutr..

[5] German J.B., Gibson R.A., Krauss R.M., Nestel P., Lamarche B., van Staveren W.A., Steijns J.M., de Groot L., Lock A.L., Destaillats F. (2009). A reappraisal of the impact of dairy foods and milk fat on cardiovascular disease risk. Eur. J. Nutr..

[6] Parodi P.W. (2009). Has the association between saturated fatty acids, serum cholesterol and coronary heart disease been over emphasized?. Int. Dairy J..

[7] Chung I.-M., Kim J.-K., Lee K.-J., Son N.-Y., An M.-J., Lee J.-H., An Y.-J., Kim S.-H. (2018). Discrimination of organic milk by stable isotope ratio, vitamin E, and fatty acid profiling combined with multivariate analysis: A case study of monthly and seasonal variation in Korea for 2016–2017. Food Chem..

[8] Palmquist D.L., Beaulieu A.D., Barbano D.M. (1993). Feed and animal factors influencing milk-fat composition. J. Dairy Sci..

[9] Jensen R.G. (2002). The composition of bovine milk lipids: January 1995 to December 2000. J. Dairy Sci..

[10] Kelsey J.A., Corl B.A., Collier R.J., Bauman D.E. (2003). The effect of breed, parity, and stage of lactation on conjugated linoleic acid (CLA) in milk fat from dairy cows. J. Dairy Sci..

[11] Pešek M., Samková E., Špička J. (2006). Fatty acids and composition of their important groups in milk fat of Czech Pied cattle. Czech J. Anim. Sci..

[12] Coppa M., Ferlay A., Chassaing C., Agabriel C., Glasser F., Chilliard Y., Borreani G., Barcarolo R., Baars T., Kusche D. (2013). Prediction of bulk milk fatty acid composition based on farming practices collected through on-farm surveys. J. Dairy Sci..

[13] Foltys V., Kirchnerová K. (2017). Impact of lactation stage and milk production on milk fat fatty acids ratio. Slovak J. Anim. Sci..

[14] Soyeurt H., Dehareng F., Mayeres P., Bertozzi C., Gengler N. (2008). Variation of delta (9)-desaturase activity in dairy cattle. J. Dairy Sci..

[15] Bittante G., Cecchinato A., Schiavon S. (2018). Dairy system, parity, and lactation stage affect enteric methane production, yield, and intensity per kilogram of milk and cheese predicted from gas chromatography fatty acids. J. Dairy Sci..

[16] Kala R., Samková E., Koubová J., Hasoňová L., Kváč M., Pelikánová T., Špička J., Hanuš O. (2018). Nutritionally desirable fatty acids including CLA of cow´s milk fat explained by animal and feed factors. Acta Univ. Agric. Silvic. Mendel. Brun..

[17] Samková E., Koubová J., Hasoňová L., Hanuš O., Kala R., Kváč M., Pelikánová T., Špička J. (2018). Joint effects of breed, parity, month of lactation, and cow individuality on the milk fatty acids composition. Mljekarstvo.

[18] Samková E., Špička J., Pešek M., Pelikánová T., Hanuš O. (2012). Animal factors affecting fatty acid composition of cow milk fat: A review. S. Afr. J. Anim. Sci..

[19] Kalač P., Samková E. (2010). The effects of feeding various forages on fatty acid composition of bovine milk fat: A review. Czech J. Anim. Sci..

[20] Shingfield K.J., Reynolds C.K., Lupoli B., Toivonen V., Yurawecz M.P., Delmonte P., Griinari J.M., Grandison A.S., Beever D.E. (2005). Effect of forage type and proportion of concentrate in the diet on milk fatty acid composition in cows given sunflower oil and fish oil. Anim. Sci..

[21] Couvreur S., Hurtaud C., Lopez C., Delaby L., Peyraud J.L. (2006). The linear relationship between the proportion of fresh grass in the cow diet, milk fatty acid composition, and butter properties. J. Dairy Sci..

[22] Kudrna V., Marounek M. (2006). The influence of feeding rapeseed cake and extruded soyabean on the performance of lactating cows and the fatty acid pattern of milk. J. Anim. Feed Sci..

[23] Bobe G., Zimmerman S., Hammond E.G., Freeman A.E., Porter P.A., Luhman C.M., Beitz D.C. (2007). Butter composition and texture from cows with different milk fatty acid compositions fed fish oil or roasted soybeans. J. Dairy Sci..

[24] Cabrita A.R.J., Bessa R.J.B., Alves S.P., Dewhurst R.J., Fonseca A.J.M. (2007). Effects of dietary protein and starch on intake, milk production, and milk fatty acid profiles of dairy cows fed corn silage-based diets. J. Dairy Sci..

[25] Frelich J., Šlachta M., Hanuš O., Špička J., Samková E. (2009). Fatty acid composition of cow milk fat produced on low-input mountain farms. Czech J. Anim. Sci..

[26] Frelich J., Šlachta M., Hanuš O., Špička J., Samková E., Weglarz A., Zapletal P. (2012). Seasonal variation in fatty acid composition of cow milk in relation to the feeding system. Anim. Sci. Pap. Rep..

[27] Veselý A., Křížová L., Třináctý J., Hadrová S., Navrátilová M., Herzig I., Fišera M. (2009). Changes in fatty acid profile and iodine content in milk as influenced by the inclusion of extruded rapeseed cake in the diet of dairy cows. Czech J. Anim. Sci..

[28] Adler S.A., Jensen S.K., Govasmark E., Steinshamn H. (2013). Effect of short-term versus long-term grassland management and seasonal variation in organic and conventional dairy farming on the composition of bulk tank milk. J. Dairy Sci..

[29] Samková E., Čertíková J., Špička J., Hanuš O., Pelikánová T., Kváč M. (2014). Eighteen-carbon fatty acids in milk fat of Czech Fleckvieh and Holstein cows following feeding with fresh lucerne (*Medicago sativa* L.). Anim. Sci. Pap. Rep..

[30] Hanuš O., Křížová L., Samková E., Špička J., Kučera J., Klimešová M., Roubal P., Jedelská R. (2016). The effect of cattle breed, season and type of diet on the fatty acid profile of raw milk. Arch. Anim. Breed..

[31] Rafiee-Yarandi H., Ghorbani G.R., Alikhani M., Sadeghi-Sefidmazgi A., Drackley J.K. (2016). A comparison of the effect of soybeans roasted at different temperatures versus calcium salts of fatty acids on performance and milk fatty acid composition of mid-lactation Holstein cows. J. Dairy Sci..

[32] Křížová L., Hanuš O., Špička J., Samková E., Frelich J., Richter M., Veselý A., Roubal P. (2016). Alternative supplemental mixture for organic dairy herds to maintain desirable milk fatty acid profile throughout the indoor feeding period. Anim. Sci. Pap. Rep..

[33] Křížová L., Ryšavý J., Richter M., Veselý A., Hanuš O., Janštová B., Vorlová L., Samková E. (2017). Milk yield, milk composition, fatty acid profile and indices of milk fat quality as affected by feeding with extruded full-fat soybean. Mljekarstvo.

[34] Soyeurt H., Dardenne P., Dehareng F., Lognay G., Veselko D., Marlier M., Bertozzi C., Mayeres P., Gengler N. (2006). Estimating fatty acid content in cow milk using mid-infrared spectrometry. J. Dairy Sci..

[35] Soyeurt H., Dehareng F., Gengler N., McParland S., Wall E., Berry D.P., Coffey M., Dardenne P. (2011). Mid-infrared prediction of bovine milk fatty acids across multiple breeds, production systems, and countries. J. Dairy Sci..

[36] Coppa M., Ferlay A., Leroux C., Jestin M., Chilliard Y., Martin B., Andueza D. (2010). Prediction of milk fatty acid composition by near infrared reflectance spectroscopy. Int. Dairy J..

[37] Ferrand-Calmels M., Palhiere I., Brochard M., Leray O., Astruc J.M., Aurel M.R., Barbey S., Bouvier F., Brunschwig P., Caillatt H. (2014). Prediction of fatty acid profiles in cow, ewe, and goat milk by mid-infrared spectrometry. J. Dairy Sci..

[38] Hanuš O., Samková E., Špička J., Hasoňová L., Kala R., Klímová Z., Kopunecz P., Kopecký J. (2015). Comparison of methods used for the determination of the healthy important fatty acids of milk fat in bulk milk samples of dairy cows. Mlék. Listy Zprav..

[39] Bernard L., Bonnet M., Delavaud C., Delosiere M., Ferlay A., Fougere H., Graulet B. (2018). Milk fat globule in ruminant: Major and minor compounds, nutritional regulation and differences among species. Eur. J. Lipid Sci. Technol..

[40] Tajima K., Aminov R.I., Nagamine T., Matsui H., Nakamura M., Benno Y. (2001). Diet-dependent shifts in the bacterial population of the rumen revealed with real-time PCR. Appl. Environ. Microb..

[41] Conte G., Dimauro C., Serra A., Macciotta N.P.P., Mele M. (2018). A canonical discriminant analysis to study the association between milk fatty acids of ruminal origin and milk fat depression in dairy cows. J. Dairy Sci..

[42] Shingfield K.J., Chilliard Y., Toivonen V., Kairenius P., Givens D.I. (2008). *Trans* fatty acids and bioactive lipids in ruminant milk. Adv. Exp. Med. Biol..

[43] Patra A.K., Yu Z.T. (2012). Effects of essential oils on methane production and fermentation by, and abundance and diversity of, rumen microbial populations. Appl. Environ. Microb..

[44] Vlaeminck B., Fievez V., Demeyer D., Dewhurst R.J. (2006). Effect of forage:concentrate ratio on fatty acid composition of rumen bacteria isolated from ruminal and duodenal digesta. J. Dairy Sci..

[45] De Menezes A.B., Lewis E., O’Donovan M., O’Neill B.F., Clipson N., Doyle E.M. (2011). Microbiome analysis of dairy cows fed pasture or total mixed ration diets. FEMS Microbiol. Ecol..

[46] Samková E. (2011). Factors Affecting Fatty Acid Composition of Cow’s Milk Fat. Doctoral Thesis.

[47] Dewhurst R.J., Scollan N.D., Lee M.R.F., Ougham H.J., Humphreys M.O. (2003). Forage breeding and management to increase the beneficial fatty acid content of ruminant products. Proc. Nutr. Soc..

[48] Vanhatalo A., Kuoppala K., Toivonen V., Shingfield K.J. (2007). Effects of forage species and stage of maturity on bovine milk fatty acid composition. Eur. J. Lipid Sci. Technol..

[49] Leiber F., Kreuzer M., Nigg D., Wettstein H.R., Scheeder M.R.L. (2005). A study on the causes for the elevated n-3 fatty acids in cows’ milk of alpine origin. Lipids.

[50] Rego O.A., Rosa H.J.D., Regalo S.M., Alves S.P., Alfaia C.M.M., Prates J.A.M., Vouzela C.M., Bessa R.J.B. (2008). Seasonal changes of CLA isomers and other fatty acids of milk fat from grazing dairy herds in the Azores. J. Sci. Food Agric..

[51] Hurtaud C., Faucon F., Couvreur S., Peyraud J.L. (2010). Linear relationship between increasing amounts of extruded linseed in dairy cow diet and milk fatty acid composition and butter properties. J. Dairy Sci..

[52] Glasser F., Ferlay A., Chilliard Y. (2008). Oilseed lipid supplements and fatty acid composition of cow milk: A meta-analysis. J. Dairy Sci..

[53] Shingfield K.J., Bonnet M., Scollan N.D. (2013). Recent developments in altering the fatty acid composition of ruminant-derived foods. Animal.

[54] Siurana A., Calsamiglia S. (2016). A metaanalysis of feeding strategies to increase the content of conjugated linoleic acid (CLA) in dairy cattle milk and the impact on daily human consumption. Anim. Feed Sci. Technol..

[55] Rutkowska J., Bialek M., Bagnicka E., Jarczak J., Tambor K., Strzalkowska N., Jozwik A., Krzyzewski J., Adamska A., Rutkowska E. (2015). Effects of replacing extracted soybean meal with rapeseed cake in corn grass silage-based diet for dairy cows. J. Dairy Res..

[56] Lopes J.C., Harper M.T., Giallongo F., Oh J., Smith L., Ortega-Perez A.M., Harper S.A., Melgar A., Kniffen D.M., Fabin R.A. (2017). Effect of high-oleic-acid soybeans on production performance, milk fatty acid composition, and enteric methane emission in dairy cows. J. Dairy Sci..

[57] Stergiadis S., Leifert C., Seal C.J., Eyre M.D., Steinshamn H., Butler G. (2014). Improving the fatty acid profile of winter milk from housed cows with contrasting feeding regimes by oilseed supplementation. Food Chem..

[58] Hristov A.N., Domitrovich C., Wachter A., Cassidy T., Lee C., Shingfield K.J., Kairenius P., Davis J., Brown J. (2011). Effect of replacing solvent-extracted canola meal with high-oil traditional canola, high-oleic acid canola, or high-erucic acid rapeseed meals on rumen fermentation, digestibility, milk production, and milk fatty acid composition in lactating dairy cows. J. Dairy Sci..

[59] Komprda T. (2009). Comparison of quality and safety of organic and conventional foods. Chem. Listy.

[60] Srednicka-Tober D., Baranski M., Seal C.J., Sanderson R., Benbrook C., Steinshamn H., Gromadzka-Ostrowska J., Rembialkowska E., Skwarlo-Sonta K., Eyre M. (2016). Higher PUFA and n-3 PUFA, conjugated linoleic acid, alpha-tocopherol and iron, but lower iodine and selenium concentrations in organic milk: A systematic literature review and meta- and redundancy analyses. Br. J. Nutr..

[61] Dangour A.D., Dodhia S.K., Hayter A., Allen E., Lock K., Uauy R. (2009). Nutritional quality of organic foods: A systematic review. Am. J. Clin. Nutr..

[62] Ellis K.A., Innocent G., Grove-White D., Cripps P., McLean W.G., Howard C.V., Mihm M. (2006). Comparing the fatty acid composition of organic and conventional milk. J. Dairy Sci..

[63] Lavrenčić A., Levart A., Salobir J. (2007). Fatty acid composition of milk produced in organic and conventional dairy herds in Italy and Slovenia. Ital. J. Anim. Sci..

[64] O’Donnell A.M., Spatny K.P., Vicini J.L., Bauman D.E. (2010). Survey of the fatty acid composition of retail milk differing in label claims based on production management practices. J. Dairy Sci..

[65] Butler G., Nielsen J.H., Slots T., Seal C., Eyre M.D., Sanderson R., Leifert C. (2008). Fatty acid and fat-soluble antioxidant concentrations in milk from high- and low-input conventional and organic systems: Seasonal variation. J. Sci. Food Agric..

[66] Slots T., Butler G., Leifert C., Kristensen T., Skibsted L.H., Nielsen J.H. (2009). Potentials to differentiate milk composition by different feeding strategies. J. Dairy Sci..

[67] Larsen M.K., Nielsen J.H., Butler G., Leifert C., Slots T., Kristiansen G.H., Gustafsson A.H. (2010). Milk quality as affected by feeding regimens in a country with climatic variation. J. Dairy Sci..

[68] Palladino R.A., O’Donovan M., Murphy J.J., McEvoy M., Callan J., Boland T.M., Kenny D.A. (2009). Fatty acid intake and milk fatty acid composition of Holstein dairy cows under different grazing strategies: Herbage mass and daily herbage allowance. J. Dairy Sci..

[69] Ferlay A., Martin B., Pradel P., Coulon J.B., Chilliard Y. (2006). Influence of grass-based diets on milk fatty acid composition and milk lipolytic system in Tarentaise and Montbeliarde cow breeds. J. Dairy Sci..

[70] Schwendel B.H., Wester T.J., Morel P.C.H., Tavendale M.H., Deadman C., Shadbolt N.M., Otter D.E. (2015). Invited review: Organic and conventionally produced milk—An evaluation of influence factors on milk composition. J. Dairy Sci..

[71] Palupi E., Jayanegara A., Ploeger A., Kahl J. (2012). Comparison of nutritional quality between conventional and organic dairy products: A meta-analysis. J. Sci. Food Agric..

[72] Schroeder G.F., Delahoy J.E., Vidaurreta I., Bargo F., Gagliostro G.A., Muller L.D. (2003). Milk fatty acid composition of cows fed a total mixed ration or pasture plus concentrates replacing corn with fat. J. Dairy Sci..

[73] Dewhurst R.J., Shingfield K.J., Lee M.R.F., Scollan N.D. (2006). Increasing the concentrations of beneficial polyunsaturated fatty acids in milk produced by dairy cows in high-forage systems. Anim. Feed Sci. Technol..

[74] Barca J., Carriquiry M., Olazabal L., Fajardo M., Chilibroste P., Meikle A. (2018). Milk fatty acid profile from cows fed with mixed rations and different access time to pastureland during early lactation. J. Anim. Physiol. Anim. Nutr..

[75] Chilliard Y., Ferlay A., Mansbridge R.M., Doreau M. (2000). Ruminant milk fat plasticity: Nutritional control of saturated, polyunsaturated, *trans* and conjugated fatty acids. Ann. Zootech..

[76] Butler G., Stergiadis S., Seal C., Eyre M., Leifert C. (2011). Fat composition of organic and conventional retail milk in northeast England. J. Dairy Sci..

[77] Collomb M., Bisig W., Butikofer U., Sieber R., Bregy M., Etter L. (2008). Fatty acid composition of mountain milk from Switzerland: Comparison of organic and integrated farming systems. Int. Dairy J..

[78] Kay J.K., Weber W.J., Moore C.E., Bauman D.E., Hansen L.B., Chester-Jones H., Crooker B.A., Baumgard L.H. (2005). Effects of week of lactation and genetic selection for milk yield on milk fatty acid composition in Holstein cows. J. Dairy Sci..

[79] Mulligan F.T., O’Grady L., Rice D.A., Doherty M.L. (2006). A herd health approach to dairy cow nutrition and production diseases of the transition cow. Anim. Reprod. Sci..

[80] Stoop W.M., Bovenhuis H., Heck J.M.L., van Arendonk J.A.M. (2009). Effect of lactation stage and energy status on milk fat composition of Holstein-Friesian cows. J. Dairy Sci..

[81] Gross J., van Dorland H.A., Bruckmaier R.M., Schwarz F.J. (2011). Milk fatty acid profile related to energy balance in dairy cows. J. Dairy Res..

[82] Arfuso F., Fazio F., Levanti M., Rizzo M., Di Pietro S., Giudice E., Piccione G. (2016). Lipid and lipoprotein profile changes in dairy cows in response to late pregnancy and the early postpartum period. Arch. Anim. Breed..

[83] Walsh S.W., Williams E.J., Evans A.C.O. (2011). A review of the causes of poor fertility in high milk producing dairy cows. Anim. Reprod. Sci..

[84] Lake S.L., Weston T.R., Scholljegerdes E.J., Murrieta C.M., Alexander B.M., Rule D.C., Moss G.E., Hess B.W. (2007). Effects of postpartum dietary fat and body condition score at parturition on plasma, adipose tissue, and milk fatty acid composition of lactating beef cows. J. Anim. Sci..

[85] Roche J.R., Friggens N.C., Kay J.K., Fisher M.W., Stafford K.J., Berry D.P. (2009). Invited review: Body condition score and its association with dairy cow productivity, health, and welfare. J. Dairy Sci..

[86] Useni B.A., Muller C.J.C., Cruywagen C.W. (2018). Pre- and postpartum effects of starch and fat in dairy cows: A review. S. Afr. J. Anim. Sci..

[87] Van Knegsel A.T.M., van den Branda H., Dijkstra J., Tamminga S., Kemp B. (2005). Effect of dietary energy source on energy balance, production, metabolic disorders and reproduction in lactating dairy cattle. Reprod. Nutr. Dev..

[88] Thatcher W., Santos J.E.P., Staples C.R. (2011). Dietary manipulations to improve embryonic survival in cattle. Theriogenology.

[89] Jorritsma R., Wensing T., Kruip T.A.M., Vos P.L.A.M., Noordhuizen J.P.T.M. (2003). Metabolic changes in early lactation and impaired reproductive performance in dairy cows. Vet. Res..

[90] Grummer R.R., Mashek D.G., Hayirli A. (2004). Dry matter intake and energy balance in the transition period. Vet. Clin. N. Am.-Food Anim. Pract..

[91] Van Straten M., Shpigel N.Y., Friger M. (2008). Analysis of daily body weight of high-producing dairy cows in the first one hundred twenty days of lactation and associations with ovarian inactivity. J. Dairy Sci..

[92] Wathes D.C., Abayasekara D.R.E., Aitken R.J. (2007). Polyunsaturated fatty acids in male and female reproduction. Biol. Reprod..

[93] Drackley J.K. (1999). Biology of dairy cows during the transition period: The final frontier?. J. Dairy Sci..

[94] Vernon R.G. (2005). Lipid metabolism during lactation: A review of adipose tissue-liver interactions and the development of fatty liver. J. Dairy Res..

[95] Schulz K., Frahm J., Meyer U., Kersten S., Reiche D., Rehage J., Danicke S. (2014). Effects of prepartal body condition score and peripartal energy supply of dairy cows on postpartal lipolysis, energy balance and ketogenesis: An animal model to investigate subclinical ketosis. J. Dairy Res..

[96] Fiore E., Piccione G., Gianesella M., Pratico V., Vazzana I., Dara S., Morgante M. (2015). Serum thyroid hormone evaluation during transition periods in dairy cows. Arch. Anim. Breed..

[97] Vranković L., Aladrović J., Octenjak D., Bijelić D., Cvetnić L., Stojević Z. (2017). Milk fatty acid composition as an indicator of energy status in Holstein dairy cows. Arch. Anim. Breed..

[98] Bauman D.E., Mather I.H., Wall R.J., Lock A.L. (2006). Major advances associated with the biosynthesis of milk. J. Dairy Sci..

[99] Stádník L., Ducháček J., Okrouhlá M., Ptáček M., Beran J., Stupka R., Zita L. (2013). The effect of parity on the proportion of important healthy fatty acids in raw milk of Holstein cows. Mljekarstvo.

[100] Garnsworthy P.C., Masson L.L., Lock A.L., Mottram T.T. (2006). Variation of milk citrate with stage of lactation and de novo fatty acid synthesis in dairy cows. J. Dairy Sci..

[101] Mele M., Dal Zotto R., Cassandro M., Conte G., Serra A., Buccioni A., Bittante G., Secchiari P. (2009). Genetic parameters for conjugated linoleic acid, selected milk fatty acids, and milk fatty acid unsaturation of Italian Holstein-Friesian cows. J. Dairy Sci..

[102] Wang T., Oh J.J., Lim J.N., Hong J.E., Kim J.H., Kim J.H., Kang H.S., Choi Y.J., Lee H.G. (2013). Effects of lactation stage and individual performance on milk *cis*-9, *trans*-11 conjugated linoleic acids content in dairy cows. Asian Australas. J. Anim..

[103] Gottardo P., Penasa M., Righi F., Lopez-Villalobos N., Cassandro M., De Marchi M. (2017). Fatty acid composition of milk from Holstein-Friesian, Brown Swiss, Simmental and Alpine Grey cows predicted by mid-infrared spectroscopy. Ital. J. Anim. Sci..

[104] Rukkwamsuk T., Geelen M.J.H., Kruip T.A.M., Wensing T. (2000). Interrelation of fatty acid composition in adipose tissue, serum, and liver of dairy cows during the development of fatty liver postpartum. J. Dairy Sci..

[105] Tyburczy C., Lock A.L., Dwyer D.A., Destaillats F., Mouloungui Z., Candy L., Bauman D.E. (2008). Uptake and utilization of *trans* octadecenoic acids in lactating dairy cows. J. Dairy Sci..

[106] Smith T.R., McNamara J.P. (1990). Regulation of bovine adipose-tissue metabolism during lactation. 6. Cellularity and hormone-sensitive lipase activity as affected by genetic merit and energy-intake. J. Dairy Sci..

[107] Van Haelst Y.N.T., Beeckman A., Van Knegsel A.T.M., Fievez V. (2008). Short communication: Elevated concentrations of oleic acid and long-chain fatty acids in milk fat of multiparous subclinical ketotic cows. J. Dairy Sci..

[108] Pedron O., Cheli F., Senatore E., Baroli D., Rizzi R. (1993). Effect of body condition score at calving on performance, some blood parameters, and milk fatty acid composition in dairy cows. J. Dairy Sci..

[109] Pires J.A.A., Delavaud C., Faulconnier Y., Pomies D., Chilliard Y. (2013). Effects of body condition score at calving on indicators of fat and protein mobilization of periparturient Holstein-Friesian cows. J. Dairy Sci..

[110] Pineyrua J.T.M., Farina S.R., Mendoza A. (2018). Effects of parity on productive, reproductive, metabolic and hormonal responses of Holstein cows. Anim. Reprod. Sci..

[111] Cavestany D., Blanc J.E., Kulcsar M., Uriarte G., Chilibroste P., Meikle A., Febel H., Ferraris A., Krall E. (2005). Studies of the transition cow under a pasture-based milk production system: Metabolic profiles. J. Vet. Med. A Physiol. Pathol. Clin. Med..

[112] Meikle A., Kulcsar M., Chilliard Y., Febel H., Delavaud C., Cavestany D., Chilibroste P. (2004). Effects of parity and body condition at parturition on endocrine and reproductive parameters of the cow. Reproduction.

[113] Wathes D.C., Cheng Z., Bourne N., Taylor V.J., Coffey M.P., Brotherstone S. (2007). Differences between primiparous and multiparous dairy cows in the inter-relationships between metabolic traits, milk yield and body condition score in the periparturient period. Domest. Anim. Endocrinol..

[114] Williams C.M. (2000). Dietary fatty acids and human health. Ann. Zootech..

[115] Belury M.A. (2002). Dietary conjugated linoleic acid in health: Physiological effects and mechanisms of action. Annu. Rev. Nutr..

[116] Auldist M.J., Walsh B.J., Thomson N.A. (1998). Seasonal and lactational influences on bovine milk composition in New Zealand. J. Dairy Res..

[117] Artegoitia V., Meikle A., Olazabal L., Damian J.P., Adrien M.L., Mattiauda D.A., Bermudez J., Torre A., Carriquiry M. (2013). Milk casein and fatty acid fractions in early lactation are affected by nutritional regulation of body condition score at the beginning of the transition period in primiparous and multiparous cows under grazing conditions. J. Anim. Physiol. Anim. Nutr..

[118] Stádník L., Ducháček J., Toušová R., Beran J., Ptáček M., Kouřimská L. (2015). Relations between basic milk components and free fatty acid content in Holstein cow milk as lipolysis parameter. Mljekarstvo.

[119] Bastin C., Soyeurt H., Gengler N. (2013). Genetic parameters of milk production traits and fatty acid contents in milk for Holstein cows in parity 1–3. J. Anim. Breed. Genet..

[120] Penasa M., Tiezzi F., Gottardo P., Cassandro M., De Marchi M. (2015). Genetics of milk fatty acid groups predicted during routine data recording in Holstein dairy cattle. Livest. Sci..

[121] Schennink A., Stoop W.M., Visker M.H.P.W., Heck J.M.L., Bovenhuis H., van der Poel J.J., van Valenberg H.J.F., van Arendonk J.A.M. (2007). *DGAT1* underlies large genetic variation in milk-fat composition of dairy cows. Anim. Genet..

[122] Garnsworthy P.C., Feng S., Lock A.L., Royal M.D. (2010). Short communication: Heritability of milk fatty acid composition and stearoyl-CoA desaturase indices in dairy cows. J. Dairy Sci..

[123] Hein L., Sorensen L.P., Kargo M., Buitenhuis A.J. (2018). Genetic analysis of predicted fatty acid profiles of milk from Danish Holstein and Danish Jersey cattle populations. J. Dairy Sci..

[124] Bastin C., Gengler N., Soyeurt H. (2011). Phenotypic and genetic variability of production traits and milk fatty acid contents across days in milk for Walloon Holstein first-parity cows. J. Dairy Sci..

[125] Petrini J., Iung L.H.S., Rodriguez M.A.P., Salvian M., Pertille F., Rovadoscki G.A., Cassoli L.D., Coutinho L.L., Machado P.F., Wiggans G.R. (2016). Genetic parameters for milk fatty acids, milk yield and quality traits of a Holstein cattle population reared under tropical conditions. J. Anim. Breed. Genet..

[126] Krag K., Poulsen N.A., Larsen M.K., Larsen L.B., Janss L.L., Buitenhuis B. (2013). Genetic parameters for milk fatty acids in Danish Holstein cattle based on SNP markers using a Bayesian approach. BMC Genet..

[127] Edwards R.A., King J.W.B., Yousef I.M. (1973). Genetic variation in fatty acid composition of cow milk. Anim. Prod..

[128] Renner E., Kosmack U. (1974). Genetische Aspekte zur Fettsäurenzusammensetzung des Milchfettes. 2. Fettsäurenmuster der Milch von Nachtkommenpopulationen. Züchtungskunde.

[129] Vanrobays M.L., Bastin C., Vandenplas J., Hammami H., Soyeurt H., Vanlierde A., Dehareng F., Froidmont E., Gengler N. (2016). Changes throughout lactation in phenotypic and genetic correlations between methane emissions and milk fatty acid contents predicted from milk mid-infrared spectra. J. Dairy Sci..

[130] Narayana S.G., Schenkel F.S., Fleming A., Koeck A., Malchiodi F., Jamrozik J., Johnston J., Sargolzaei M., Miglior F. (2017). Genetic analysis of groups of mid-infrared predicted fatty acids in milk. J. Dairy Sci..

[131] Mosley E.E., Shafii B., Moate P.J., McGuire M.A. (2006). *cis*-9, *trans*-11 conjugated linoleic acid is synthesized directly from vaccenic acid in lactating dairy cattle. J. Nutr..

[132] Bobe G., Bormann J.A.M., Lindberg G.L., Freeman A.E., Beitz D.C. (2008). Short communication: Estimates of genetic variation of milk fatty acids in US Holstein cows. J. Dairy Sci..

[133] Poulsen N.A., Eskildsen C.E.A., Skov T., Larsen L.B., Buitenhuis A.J. Comparison of genetic parameters estimation of fatty acids from gas chromatography and FT-IR in Holsteins. Proceedings of the 10th World Congress of Genetics Applied to Livestock Production.

[134] Soyeurt H., Dardenne P., Dehareng F., Bastin C., Gengler N. (2008). Genetic parameters of saturated and monounsaturated fatty acid content and the ratio of saturated to unsaturated fatty acids in bovine milk. J. Dairy Sci..

[135] Stoop W.M., van Arendonk J.A.M., Heck J.M.L., van Valenberg H.J.F., Bovenhuis H. (2008). Genetic parameters for major milk fatty acids and milk production traits of Dutch Holstein-Friesians. J. Dairy Sci..

[136] Bastin C., Soyeurt H., Vanderick S., Gengler N. (2011). Genetic relationships between milk fatty acids and fertility of dairy cows. Interbull Bull..

[137] Lassen J., Poulsen N.A., Larsen M.K., Buitenhuis A.J. (2016). Genetic and genomic relationship between methane production measured in breath and fatty acid content in milk samples from Danish Holsteins. Anim. Prod. Sci..

[138] Qanbari S., Pimentel E.C.G., Tetens J., Thaller G., Lichtner P., Sharifi A.R., Simianer H. (2010). A genome-wide scan for signatures of recent selection in Holstein cattle. Anim. Genet..

[139] Bouwman A.C., Bovenhuis H., Visker M.H.P.W., van Arendonk J.A.M. (2011). Genome-wide association of milk fatty acids in Dutch dairy cattle. BMC Genet..

[140] Bouwman A.C., Visker M.H.P.W., van Arendonk J.A.M., Bovenhuis H. (2012). Genomic regions associated with bovine milk fatty acids in both summer and winter milk samples. BMC Genet..

[141] Buitenhuis B., Janss L.L.G., Poulsen N.A., Larsen L.B., Larsen M.K., Sorensen P. (2014). Genome-wide association and biological pathway analysis for milk-fat composition in Danish Holstein and Danish Jersey cattle. BMC Genom..

[142] Li C., Sun D.X., Zhang S.L., Yang S.H., Alim M.A., Zhang Q., Li Y.H., Liu L. (2016). Genetic effects of *FASN*, *PPARGC1A, ABCG2* and *IGF1* revealing the association with milk fatty acids in a Chinese Holstein cattle population based on a post genome-wide association study. BMC Genet..

[143] Lopdell T.J., Tiplady K., Struchalin M., Johnson T.J.J., Keehan M., Sherlock R., Couldrey C., Davis S.R., Snell R.G., Spelman R.J. (2017). DNA and RNA-sequence based GWAS highlights membrane-transport genes as key modulators of milk lactose content. BMC Genom..

[144] Bionaz M., Loor J.J. (2008). Gene networks driving bovine milk fat synthesis during the lactation cycle. BMC Genom..

[145] Conte G., Mele M., Chessa S., Castiglioni B., Serra A., Pagnacco G., Secchiari P. (2010). Diacylglycerol acyltransferase 1, stearoyl-CoA desaturase 1, and sterol regulatory element binding protein 1 gene polymorphisms and milk fatty acid composition in Italian Brown cattle. J. Dairy Sci..

[146] Bovenhuis H., Visker M.H.P.W., Poulsen N.A., Sehested J., van Valenberg H.J.F., van Arendonk J.A.M., Larsen L.B., Buitenhuis A.J. (2016). Effects of the diacylglycerol o-acyltransferase 1 (DGAT1) K232A polymorphism on fatty acid, protein, and mineral composition of dairy cattle milk. J. Dairy Sci..

[147] Schennink A., Heck J.M.L., Bovenhuis H., Visker M.H.P.W., van Valenberg H.J.F., van Arendonk J.A.M. (2008). Milk fatty acid unsaturation: Genetic parameters and effects of stearoyl-CoA desaturase (SCD1) and acyl CoA: Diacylglycerol acyltransferase 1 (DGAT1). J. Dairy Sci..

[148] Pešek M., Špička J., Samková E. (2005). Comparison of fatty acid composition in milk fat of Czech Pied cattle and Holstein cattle. Czech J. Anim. Sci..

[149] Soyeurt H., Dardenne P., Gillon A., Croquet C., Vanderick S., Mayeres P., Bertozzi C., Gengler N. (2006). Variation in fatty acid contents of milk and milk fat within and across breeds. J. Dairy Sci..

[150] Soyeurt H., Gillon A., Vanderick S., Mayeres P., Bertozzi C., Gengler N. (2007). Estimation of heritability and genetic correlations for the major fatty acids in bovine milk. J. Dairy Sci..

[151] Pilarczyk R., Wójcik J., Sablik P., Czerniak P. (2015). Fatty acid profile and health lipid indices in the raw milk of Simmental and Holstein-Friesian cows from an organic farm. S. Afr. J. Anim. Sci..

[152] Morales M.S., Palmquist D.L., Weiss W.P. (2000). Milk fat composition of Holstein and Jersey cows with control or depleted copper status and fed whole soybeans or tallow. J. Dairy Sci..

[153] White S.L., Bertrand J.A., Wade M.R., Washburn S.P., Green J.T., Jenkins T.C. (2001). Comparison of fatty acid content of milk from Jersey and Holstein cows consuming pasture or a total mixed ration. J. Dairy Sci..

[154] Bargo F., Delahoy J.E., Schroeder G.F., Baumgard L.H., Muller L.D. (2006). Supplementing total mixed rations with pasture increase the content of conjugated linoleic acid in milk. Anim. Feed Sci. Technol..

[155] Croissant A.E., Washburn S.P., Dean L.L., Drake M.A. (2007). Chemical properties and consumer perception of fluid milk from conventional and pasture-based production systems. J. Dairy Sci..

[156] Mäntysaari P., Khalili H., Sariola J., Rantanen A. (2007). Use of barley fibre and wet distillers’ solubles as feedstuffs for Ayrshire dairy cows. Anim. Feed Sci. Technol..

[157] Moioli B., Contarini G., Avalli A., Catillo G., Orru L., De Matteis G., Masoero G., Napolitano F. (2007). Short communication: Effect of stearoyl-coenzyme A desaturase polymorphism on fatty acid composition of milk. J. Dairy Sci..

[158] Barłowska J., Grodzicki T., Topyła B., Litwińczuk Z. (2009). Physicochemical properties of milk fat from three breeds of cows during summer and winter feeding. Arch. Tierzucht.

[159] Adamska A., Rutkowska J., Tabaszewska M. (2014). Milk of Polish Red and White cows as a source of nutritionally valuable fatty acids. Arch. Tierzucht.

[160] Kulig H., Kowalewska-Luczak I., Kmiec M., Wojdak-Maksymiec K. (2010). *ANXA9, SLC27A3, FABP3* and *FABP4* single nucleotide polymorphisms in relation to milk production traits in Jersey cows. Czech J. Anim. Sci..

[161] Tomka J., Vasickova K., Oravcova M., Bauer M., Huba J., Vasicek D., Peskovicova D. (2016). Effects of polymorphisms in *DGAT1* and *LEP* genes on milk traits in Holstein primiparous cows. Mljekarstvo.

[162] Cohen-Zinder M., Seroussi E., Larkin D.M., Loor J.J., Everts-van der Wind A., Lee J.H., Drackley J.K., Band M.R., Hernandez A.G., Shani M. (2005). Identification of a missense mutation in the bovine *ABCG2* gene with a major effect on the QTL on chromosome 6 affecting milk yield and composition in Holstein cattle. Genome Res..

[163] Weikard R., Kuhn C., Goldammer T., Freyer G., Schwerin M. (2005). The bovine *PPARGC1A* gene: Molecular characterization and association of an SNP with variation of milk fat synthesis. Physiol. Genom..

[164] Khatib H., Zaitoun I., Wiebelhaus-Finger J., Chang Y.M., Rosa G.J.M. (2007). The association of bovine *PPARGC1A* and *OPN* genes with milk composition in two independent holstein cattle populations. J. Dairy Sci..

[165] Grisart B., Coppieters W., Farnir F., Karim L., Ford C., Berzi P., Cambisano N., Mni M., Reid S., Simon P. (2002). Positional candidate cloning of a QTL in dairy cattle: Identification of a missense mutation in the bovine *DGAT1* gene with major effect on milk yield and composition. Genome Res..

[166] Matsumoto H., Inada S., Kobayashi E., Abe T., Hasebe H., Sasazaki S., Oyama K., Mannen H. (2012). Identification of SNPs in the *FASN* gene and their effect on fatty acid milk composition in Holstein cattle. Livest. Sci..

[167] Yao J.B., Aggrey S.E., Zadworny D., Hayes J.F., Kühnlein U. (1996). Sequence variations in the bovine growth hormone gene characterized by single-strand conformation polymorphism (SSCP) analysis and their association with milk production traits in Holsteins. Genetics.

[168] He X., Chu M.X., Qiao L., He J.N., Wang P.Q., Feng T., Di R., Cao G.L., Fang L., An Y.F. (2012). Polymorphisms of *STAT5A* gene and their association with milk production traits in Holstein cows. Mol. Biol. Rep..

[169] Nafikov R.A., Schoonmaker J.R., Korn K.T., Noack K., Garrick D.J., Koehler K.J., Minick-Bormann J., Reecy J.M., Spurlock D.E., Beitz D.C. (2013). Sterol regulatory element binding transcription factor 1 (*SREBF1*) polymorphism and milk fatty acid composition. J. Dairy Sci..

[170] Waters S.M., McCabe M.S., Howard D.J., Giblin L., Magee D.A., MacHugh D.E., Berry D.P. (2011). Associations between newly discovered polymorphisms in the *Bos taurus* growth hormone receptor gene and performance traits in Holstein-Friesian dairy cattle. Anim. Genet..

[171] Littlejohn M.D., Tiplady K., Lopdell T., Law T.A., Scott A., Harland C., Sherlock R., Henty K., Obolonkin V., Lehnert K. (2014). Expression Variants of the lipogenic *AGPAT6* gene affect diverse milk composition phenotypes in *Bos taurus*. PLoS ONE.

[172] Soyeurt H., Gengler N. (2008). Genetic variability of fatty acids in bovine milk. Biotechnol. Agron. Soc..

[173] Ulbricht T.L.V., Southgate D.A.T. (1991). Coronary heart disease—7 dietary factors. Lancet.

[174] Santos-Silva J., Mendes I.A., Bessa R.J.B. (2002). The effect of genotype, feeding system and slaughter weight on the quality of light lambs—1. Growth, carcass composition and meat quality. Livest. Prod. Sci..

[175] Chen S., Bobe G., Zimmerman S., Hammond E.G., Luhman C.M., Boylston T.D., Freeman A.E., Beitz D.C. (2004). Physical and sensory properties of dairy products from cows with various milk fatty acid compositions. J. Agric. Food Chem..

[176] Ntambi J.M., Miyazaki M. (2004). Regulation of stearoyl-CoA desaturases and role in metabolism. Prog. Lipid Res..

[177] Lock A.L., Garnsworthy P.C. (2003). Seasonal variation in milk conjugated linoleic acid and Delta(9)-desaturase activity in dairy cows. Livest. Prod. Sci..

[178] Chouinard P.Y., Corneau L., Barbano D.M., Metzger L.E., Bauman D.E. (1999). Conjugated linoleic acids alter milk fatty acid composition and inhibit milk fat secretion in dairy cows. J. Nutr..

[179] Mele M., Conte G., Castiglioni B., Chessa S., Macciotta N.P.P., Serra A., Buccioni A., Pagnacco G., Secchiari P. (2007). Stearoyl-coenzyme A desaturase gene polymorphism and milk fatty acid composition in Italian Holsteins. J. Dairy Sci..

[180] Erickson M.C. (1992). Variation of lipid and tocopherol composition in 3 strains of channel catfish (*Ictalurus punctatus*). J. Sci. Food Agric..

[181] Saito M., Kubo K. (2003). Relationship between tissue lipid peroxidation and peroxidizability index after alpha-linolenic, eicosapentaenoic, or docosahexaenoic acid intake in rats. Br. J. Nutr..

[182] Nagyová A., Krajčovičová-Kudláčková M., Klvanová J. (2001). LDL and HDL oxidation and fatty acid composition in vegetarians. Ann. Nutr. Metab..

[183] Timmen H. (1990). Characterization of milk fat hardness in farm milk via parameters of fatty-acid composition. Kieler Milchw. Forsch..

[184] Hurtaud C., Buchin S., Martin B., Verdier-Metz I., Peyraud J.L., Noel Y. (2001). Milk quality and consequences on quality of dairy products: Several some measuring techniques of measure in dairy cows trials. Recontres autour des Recherches sur Ruminant.

[185] Hanuš O., Samková E., Špička J., Sojková K., Hanušová K., Kopec T., Vyletělová M., Jedelská R. (2010). Relationship between concentration of health important groups of fatty acids and components and technological properties in cow milk. Acta Univ. Agric. Silvic. Mendel. Brun..

[186] Brzozowska A.M., Lukaszewicz M., Oprazadek J.M. (2018). Energy-protein supplementation and lactation affect fatty acid profile of liver and adipose tissue of dairy cows. Molecules.

[187] Drewnowski A. (2011). The contribution of milk and milk products to micronutrient density and affordability of the US diet. J. Am. Coll. Nutr..

[188] Haug A., Hostmark A.T., Harstad O.M. (2007). Bovine milk in human nutrition—A review. Lipids Health Dis..

[189] Mills S., Ross R.P., Hill C., Fitzgerald G.F., Stanton C. (2011). Milk intelligence: Mining milk for bioactive substances associated with human health. Int. Dairy J..

[190] Jenkins T.C., McGuire M.A. (2006). Major advances in nutrition: Impact on milk composition. J. Dairy Sci..

[191] Givens D.I. (2012). Symposium 1: Food chain and health milk in the diet: Good or bad for vascular disease?. Proc. Nutr. Soc..

[192] Kromhout D., Bloemberg B., Feskens E., Menotti A., Nissinen A., Grp S.C.S. (2000). Saturated fat, vitamin C and smoking predict long-term population all-cause mortality rates in the Seven Countries Study. Int. J. Epidemiol..

[193] Simopoulos A.P. (2002). The importance of the ratio of omega-6/omega-3 essential fatty acids. Biomed. Pharmacother..

[194] Calder P.C. (2015). Functional roles of fatty acids and their effects on human health. J. Parenter. Enter. Nutr..

[195] Wong J.M.W., de Souza R., Kendall C.W.C., Emam A., Jenkins D.J.A. (2006). Colonic health: Fermentation and short chain fatty acids. J. Clin. Gastroenterol..

[196] Van der Beek C.M., Dejong C.H.C., Troost F.J., Masclee A.M., Lenaerts K. (2017). Role of short-chain fatty acids in colonic inflammation, carcinogenesis, and mucosal protection and healing. Nutr. Rev..

[197] Meijer K., de Vos P., Priebe M.G. (2010). Butyrate and other short-chain fatty acids as modulators of immunity: What relevance for health?. Curr. Opin. Clin. Nutr..

[198] Arnould V.M.R., Soyeurt H. (2009). Genetic variability of milk fatty acids. J. Appl. Genet..

[199] Yang Z.H., Liu S.P., Chen X.D., Chen H., Huang M., Zheng J.P. (2000). Induction of apoptotic cell death and in vivo growth inhibition of human cancer cells by a saturated branched-chain fatty acid, 13-methyltetradecanoic acid. Cancer Res..

[200] Cai Q.Q., Huang H.Q., Qian D., Chen K.L., Luo J.H., Tian Y., Lin T.X., Lin T.Y. (2013). 13-Methyltetradecanoic acid exhibits anti-tumor activity on T-cell lymphomas in vitro and in vivo by down-regulating p-AKT and activating caspase-3. PLoS ONE.

[201] Wongtangtintharn S., Oku H., Iwasaki H., Toda T. (2004). Effect of branched-chain fatty acids on fatty acid biosynthesis of human breast cancer cells. J. Nutr. Sci. Vitaminol..

[202] Ran-Ressler R.R., Khailova L., Arganbright K.M., Adkins-Rieck C.K., Jouni Z.E., Koren O., Ley R.E., Brenna J.T., Dvorak B. (2011). Branched chain fatty acids reduce the incidence of necrotizing *Enterocolitis* and alter gastrointestinal microbial ecology in a neonatal rat model. PLoS ONE.

[203] Kraft J., Jetton T., Satish B., Gupta D. (2015). Dairy-derived bioactive fatty acids improve pancreatic beta-cell function. FASEB J..

[204] Khaw K.T., Friesen M.D., Riboli E., Luben R., Wareham N. (2012). Plasma phospholipid fatty Acid concentration and incident coronary heart disease in men and women: The EPIC-Norfolk prospective study. PLoS Med..

[205] Forouhi N.G., Koulman A., Sharp S.J., Imamura F., Kroger J., Schulze M.B., Crowe F.L., Huerta J.M., Guevara M., Beulens J.W.J. (2014). Differences in the prospective association between individual plasma phospholipid saturated fatty acids and incident type 2 diabetes: The EPIC-InterAct case-cohort study. Lancet Diabetes Endocrinol..

[206] Weggemans R.M., Rudrum M., Trautwein E.A. (2004). Intake of ruminant versus industrial *trans* fatty acids and risk of coronary heart disease—What is the evidence?. Eur. J. Lipid Sci. Technol..

[207] Lock A.L., Parodi P.W., Bauman D.E. (2005). The biology of *trans* fatty acids: Implications for human health and the dairy industry. Aust. J. Dairy Technol..

[208] Bendsen N.T., Christensen R., Bartels E.M., Astrup A. (2011). Consumption of industrial and ruminant *trans* fatty acids and risk of coronary heart disease: A systematic review and meta-analysis of cohort studies. Eur. J. Clin. Nutr..

[209] Anadon A., Martinez-Larranaga M.R., Martinez M.A., Ares I., Ramos E., Gomez-Cortes P., Juarez M., De la Fuente M.A. (2010). Acute oral safety study of dairy fat rich in *trans*-10 C18:1 versus vaccenic plus conjugated linoleic acid in rats. Food Chem. Toxicol..

[210] Anadon A., Martinez-Larranaga M.R., Martinez M.A., Ares I., Ramos E., Gomez-Cortes P., Juarez M., de la Fuente M.A. (2011). A 4-week repeated oral dose toxicity study of dairy fat naturally enriched in vaccenic, rumenic and alpha-linolenic acids in rats. J. Agric. Food Chem..

[211] De Souza R.J., Mente A., Maroleanu A., Cozma A.I., Ha V., Kishibe T., Uleryk E., Budylowski P., Schunemann H., Beyene J. (2015). Intake of saturated and *trans* unsaturated fatty acids and risk of all cause mortality, cardiovascular disease, and type 2 diabetes: Systematic review and meta-analysis of observational studies. BMJ..

[212] Ferlay A., Bernard L., Meynadier A., Malpuech-Brugere C. (2017). Production of *trans* and conjugated fatty acids in dairy ruminants and their putative effects on human health: A review. Biochimie.

[213] Kennedy A., Martinez K., Chuang C.C., LaPoint K., McIntosh M. (2009). Saturated fatty acid-mediated inflammation and insulin resistance in adipose tissue: Mechanisms of action and implications. J. Nutr..

[214] Kennedy A., Martinez K., Chung S., LaPoint K., Hopkins R., Schmidt S.F., Andersen K., Mandrup S., McIntosh M. (2010). Inflammation and insulin resistance induced by *trans*-10, *cis*-12 conjugated linoleic acid depend on intracellular calcium levels in primary cultures of human adipocytes. J. Lipid Res..

[215] Moon H.S. (2014). Biological effects of conjugated linoleic acid on obesity-related cancers. Chem.-Biol. Interact..

[216] Field C.J., Blewett H.H., Proctor S., Vine D. (2009). Human health benefits of vaccenic acid. Appl. Physiol. Nutr. Metab..

[217] Frigolet M.E., Gutierrez-Aguilar R. (2017). The role of the novel lipokine palmitoleic acid in health and disease. Adv. Nutr..

[218] Muchenje V., Dzama K., Chimonyo M., Strydom P.E., Hugo A., Raats J.G. (2009). Some biochemical aspects pertaining to beef eating quality and consumer health: A review. Food Chem..

[219] Zhao G.X., Etherton T.D., Martin K.R., West S.G., Gillies P.J., Kris-Etherton P.M. (2004). Dietary alpha-linolenic acid reduces inflammatory and lipid cardiovascular risk factors in hypercholesterolemic men and women. J. Nutr..

[220] Liu J.J., Ma D.W.L. (2014). The Role of n-3 polyunsaturated fatty acids in the prevention and treatment of breast cancer. Nutrients.

[221] Destaillats F., Trottier J.P., Galvez J.M.G., Angers P. (2005). Analysis of alpha-linolenic acid biohydrogenation intermediates in milk fat with emphasis on conjugated linolenic acids. J. Dairy Sci..

[222] Lerch S., Shingfield K.J., Ferlay A., Vanhatalo A., Chilliard Y. (2012). Rapeseed or linseed in grass-based diets: Effects on conjugated linoleic and conjugated linolenic acid isomers in milk fat from Holstein cows over 2 consecutive lactations. J. Dairy Sci..

[223] Allen B.G., Bhatia S.K., Anderson C.M., Eichenberger-Gilmore J.M., Sibenaller Z.A., Mapuskar K.A., Schoenfeld J.D., Buatti J.M., Spitz D.R., Fath M.A. (2014). Ketogenic diets as an adjuvant cancer therapy: History and potential mechanism. Redox Biol..

[224] Lamarche B., Givens D.I., Soedamah-Muthu S., Krauss R.M., Jakobsen M.U., Bischoff-Ferrari H.A., Pan A., Despres J.P. (2016). Does milk consumption contribute to cardiometabolic health and overall diet quality?. Can. J. Cardiol..

[225] Kiczorowska B., Samolinska W., Marczuk J., Winiarska-Mieczan A., Klebaniuk R., Kowalczuk-Vasilev E., Kiczorowski P., Zasadna Z. (2017). Comparative effects of organic, traditional, and intensive production with probiotics on the fatty acid profile of cow’s milk. J. Food Compos. Anal..

[226] Lukiw W.J., Cui J.G., Marcheselli V.L., Bodker M., Botkjaer A., Gotlinger K., Serhan C.N., Bazan N.G. (2005). A role for docosahexaenoic acid-derived neuroprotectin D1 in neural cell survival and Alzheimer disease. J. Clin. Investig..

